# Decouple H_2_O_2_ Electrosynthesis From Microenvironmental Decomposition via Atomic Site Density Engineering

**DOI:** 10.1002/anie.8125244

**Published:** 2026-08-05

**Authors:** Junwen Chen, Hongyu Zhou, Qiming Zhang, Shuang Zhong, Wei Ren, Lei Shi, Li Gao, Shaobin Wang, Xiaoguang Duan

**Affiliations:** ^1^ School of Chemical Engineering Adelaide University Adelaide Australia; ^2^ College of Materials Science and Engineering Nanjing Forestry University Nanjing China; ^3^ School of Science, STEM College RMIT University Melbourne Australia

**Keywords:** electrified membrane, hydrogen peroxide electrosynthesis, single‐atom catalysts, site density engineering, two‐electron oxygen reduction

## Abstract

Electrocatalytic H_2_O_2_ production through two‐electron oxygen reduction reaction (2e^−^‐ORR) offers a promising route to decentralised chemical synthesis and water purification. However, Faradaic efficiency typically falls short of intrinsic selectivity due to competing H_2_O_2_ reduction reaction (HPRR) and disproportionation reaction (HDR). Here, we show that active atomic density engineering governs this microenvironmental loss and can secure the net H_2_O_2_ output. Using a series of defect‐rich Co‐N_2_O_2_ single‐atom catalysts (SACs), closely spaced sites induce inter‐site electronic coupling and broaden the d‐band, which promotes further reaction with the freshly produced H_2_O_2_ toward reduction and disproportionation. Isolating the sites at an optimal Co loading of 0.84 wt.% (Co/NOC‐0.8) effectively suppresses side reactions by increasing the thermodynamic barrier to OH*/O* formation, protecting H_2_O_2_ from secondary dissociation. The resulting catalyst reduces secondary H_2_O_2_ consumption by up to 79% and closes the gap between intrinsic selectivity (86.5%) and practical Faradaic efficiency (78.7%). When integrated into a flow‐through bilayer electrified membrane reactor, the optimised catalyst combines convection‐enhanced mass transport with rapid product release at the isolated sites, achieving over 90% single‐pass removal of aniline for more than 72 h. These results define a site‐density principle for balancing product formation and product preservation in single‐atom electrocatalysis.

## Graphical abstract

Using defect‐rich Co‐N_2_O_2_ single‐atom catalysts as a model system, this work reveals that atomic site density governs the competition between H_2_O_2_ electrosynthesis and microenvironmental decomposition during 2e^−^‐ORR. Optimised site isolation suppresses HPRR and HDR, thereby establishing a site‐density principle to balance H_2_O_2_ formation and preservation.

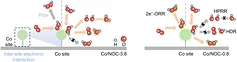

## 1 Introduction

Hydrogen peroxide (H_2_O_2_) is a versatile green oxidant used widely in chemical synthesis, energy conversion, and water treatment [1]. Compared with the energy‐intensive anthraquinone process, electrocatalytic production through the two‐electron oxygen reduction reaction (2e^−^‐ORR) provides a promising route to decentralised, c H_2_O_2_ synthesis under mild conditions [2]. Most studies to date have focused on the intrinsic selectivity between the 2e^−^‐ORR and 4e^−^‐ORR pathways, which is sensitive to the chemical configuration and electronic state of the active centre [3, 4, 5, 6]. In practice, however, the Faradaic efficiency (FE) measured during H_2_O_2_ production falls well below this intrinsic selectivity, indicating that high intrinsic selectivity is necessary but not sufficient for high net H_2_O_2_ accumulation. Understanding the kinetic competition between rapid H_2_O_2_ release from the electrode surface and its subsequent, largely unproductive consumption is therefore an essential and often overlooked prerequisite for efficient electrosynthesis.

Electrogenerated H_2_O_2_ is typically consumed in‐situ through two competing pathways: the electrochemical H_2_O_2_ reduction reaction (HPRR) and the H_2_O_2_ disproportionation reaction (HDR). HPRR further reduces H_2_O_2_ to H_2_O at active sites by two‐electron transfer, whereas HDR is a spontaneous, non‐electrochemical decomposition of H_2_O_2_ into H_2_O and O_2_ without external electron input [5, 6]. Two strategies are commonly used to suppress these losses: electronic modulation and geometric isolation of active sites. Interfacial strain, for example, can downshift the d‐band centre or break electronic symmetry, perturbing the scaling relationships among oxygenated intermediates and so weakening intermediate binding and raising the energy barrier to over‐reduction [5, 7]. Geometric isolation of catalytic centres can favour an end‐on O_2_ adsorption mode that kinetically hinders the O‐O bond cleavage required for both the 4e^−^‐ORR and HPRR [5]. A catalyst system with atomic precision, in which both the electronic configuration and the spatial distribution of active centres can be tuned, is therefore needed to disentangle the mechanisms that govern H_2_O_2_ production and loss and to guide selective 2e^−^ORR catalyst design.

Single‐atom catalysts (SACs) are well suited to disentangling complex catalytic pathways because of their atomic‐scale precision and structural tunability [8, 9, 10, 11, 12, 13, 14, 15, 16, 17, 18]. The electronic features of a SAC are set primarily by the coordination environment, which, in turn, governs its intrinsic catalytic behaviour [14, 19, 20]. Introducing asymmetric motifs into the first coordination shell, for example, can shift the metal *d*‐band centre and tune the oxidation state of the active centre [2, 18]. Engineering of the second coordination shell can further break the electronic symmetry of the single‐atom site, modulating the adsorption configuration and binding strength of reaction intermediates along the desired pathway [21].

Beyond the individual coordination structure, the loading density of a SAC strongly influences catalytic behaviour by setting the spatial proximity of active sites [22]. Increasing the site density is a conventional route to higher mass activity, but it also shortens inter‐site distances and induces electronic coupling and spatial synergies that reshape the reaction microenvironment. In Fe‐N_4_ systems, such proximity‐induced coupling has been shown to downshift the $d_{z^{2}}$ orbital near the Fermi level and to increase, for example, orbital filling, which weakens the binding of oxygenated intermediates (OH*) and accelerates key steps of the 4e^−^‐ORR [12]. For H_2_O_2_ electrosynthesis, by contrast, the role of inter‐site interactions involves a delicate trade‐off: high selectivity calls for moderate binding of OOH* and OH* to promote O_2_ adsorption and H_2_O_2_ formation, while a sufficient thermodynamic barrier is also needed to suppress subsequent H_2_O_2_ consumption through HPRR and HDR [19, 20, 23, 24, 25, 26]. Site proximity is expected to be a powerful handle for tuning these adsorption configurations and energies, yet its role in balancing 2e^−^‐ORR and product‐loss pathways has not been examined [1, 7, 27, 28, 29, 30, 31, 32, 33, 34]. Establishing the density‐performance relationship is therefore essential for the rational design of high‐yield H_2_O_2_ electrosynthesis catalysts.

Here, we prepare a series of defect‐rich Co‐N_2_O_2_ SACs with precisely controlled Co loadings (0.84–3.83 wt.%) to examine the interplay between atomic coordination and site proximity. A kinetic model is then used to quantify how site density affects the competition between H_2_O_2_ generation and its consumption through HPRR and HDR. In‐situ ATR‐FTIR measurements and density functional theory (DFT) calculations identify the electronic and spatial origins of the site‐isolation effect, showing how the local electronic environment of the Co centres preserves reaction intermediates and limits product loss. The practical relevance of the design is demonstrated in a crossflow bilayer electro‐Fenton membrane system that achieves efficient continuous pollutant removal. The study provides a unified design principle for high‐yield H_2_O_2_ electrosynthesis and clarifies the role of density‐dependent interactions in single‐atom catalysis.

## 2 Results and Discussion

### 2.1 Electrocatalyst Synthesis and Characterisation

A series of Co SACs with tuneable atomic densities was prepared in three steps: coordination polymerisation, pyrolytic carbonisation, and oxidation‐induced defect formation (Figure 1a; details in the Supporting Information). A 2D hydrogen‐bonded supramolecular assembly was first built from melamine and cyanuric acid, with L‐alanine and phytic acid coordinating to Co^2+^ to disperse and pre‐anchor the metal sites against aggregation (Figure S1) [17]. High‐temperature pyrolysis then drove a topotactic conversion of the molecular assembly into 2D carbon nanosheets bearing isolated Co‐N_2_O_2_ active centres (Figure S2). A final oxidation step introduced structural vacancies near these sites and modulated their electronic structure. This route provides precise control over Co loadings between 0.27 and 3.83 wt.% (denoted Co/NOC‐0.3, ‐0.5, ‐0.8, ‐2.0, and ‐3.8, corresponding to the Co contents listed in Table S1), providing a model platform for studying site‐density‐dependent catalysis.

**FIGURE 1:**
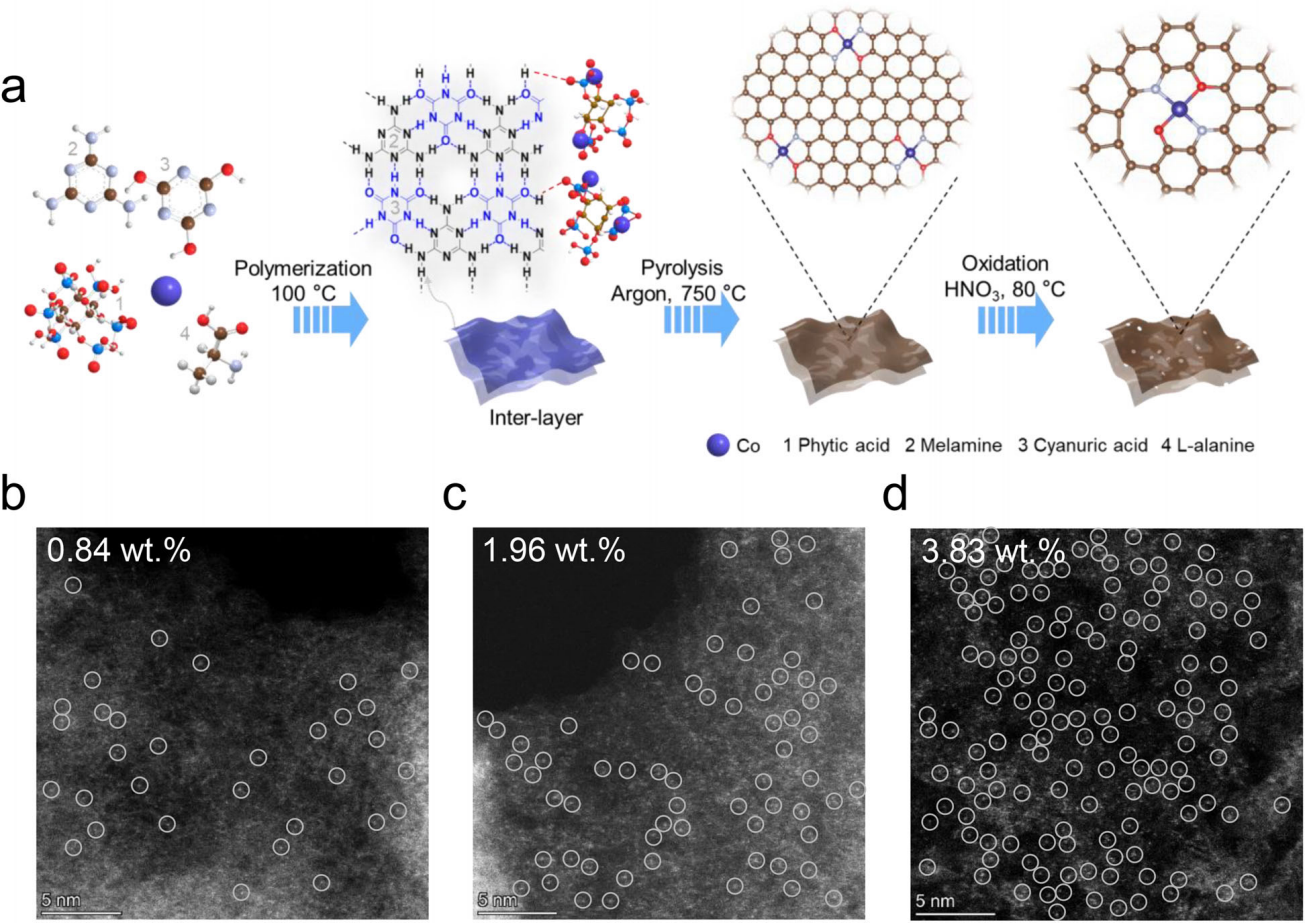
(a) Schematic illustration of the synthetic procedures. Aberration‐corrected HAADF‐STEM images of Co SACs with Co loadings of (b) 0.84 wt.%, (c)1.96 wt.%, and (d) 3.83 wt.%, respectively. Co single atoms are marked in white circles.

To examine the morphology of the Co SACs, scanning electron microscopy (SEM), transmission electron microscopy (TEM), and aberration‐corrected high‐angle annular dark‐field scanning transmission electron microscopy (HAADF‐STEM) were used. The catalysts show a hierarchically porous carbon matrix with a crumpled, multi‐layered surface (Figure S3). Elemental mapping (Figure S4) confirms that Co, N, and O are uniformly distributed throughout the porous carbon. At the atomic scale, HAADF‐STEM images (Figure 1b–d) resolve isolated Co atoms as bright dots anchored on the carbon support [35]. The spatial density of these bright dots increases progressively from Co/NOC‐0.8 to Co/NOC‐3.8, confirming controlled regulation of the atomic loading. X‐ray diffraction (XRD) patterns (Figure S5) show a single broad peak near 24°, characteristic of the amorphous carbon (002) plane, with no metal‐related reflections.

The chemical structure of the Co centres was probed by x‐ray photoelectron spectroscopy (XPS) and x‐ray absorption spectroscopy (XAS). The N 1s spectra (Figure S6) show a distinct peak at 399.3 eV assigned to the Co‐N bond, indicating a nitrogen‐rich environment around the Co sites [36]. This assignment is supported by N K‐edge XAS (Figure 2a), where the absorption peak at ∼400.3 eV, absent in the Co‐free NOC, corresponds to transitions from N 1s into hybridised N 2p‐Co 3d orbitals [37, 38, 39]. The O K‐edge XAS (Figure 2b) shows a characteristic peak at ∼530.9 eV assigned to Co‐O bonds [39, 40]. Together, these features confirm that both N and O ligands are incorporated into the first coordination shell, forming a Co‐N/O configuration. The intensities of the Co‐N and Co‐O peaks increase systematically with Co loading. The Co 2p XPS spectra (Figure S7) also show shifted binding energies across the Co/NOC series, indicating that the oxidation state of the Co centres depends on the site density [28, 41, 42, 43].

**FIGURE 2:**
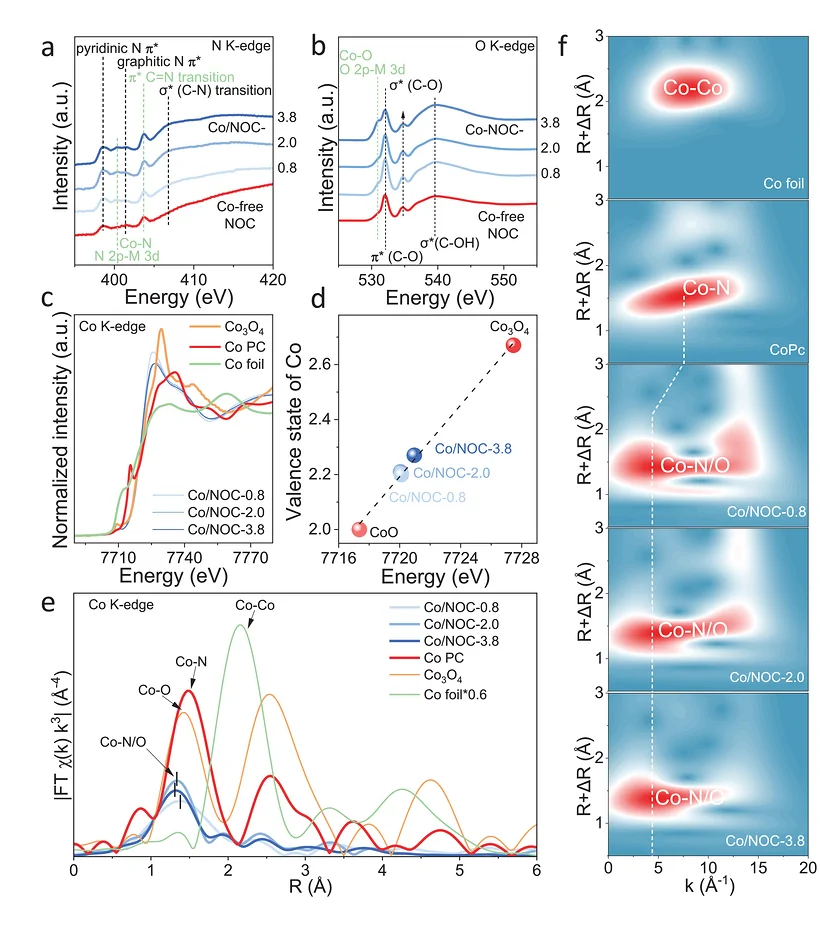
(a) N k‐edge and (b) O k‐edge XANES spectra of several Co SACs with different Co loadings. (c) X‐ray near edge structure (XANES) spectra. (d) Co valence distributions. (e) k^3^‐weighted Fourier transform (FT) EXAFS spectra. (f) Corresponding wavelet transform (WT) analysis.

The valence state and local coordination of the Co centres were further examined by x‐ray absorption fine structure (XAFS) spectroscopy. The Co K‐edge x‐ray absorption near‐edge structure (XANES) places the absorption energy of all Co/NOC samples between those of the CoO and Co_3_O_4_ references (Figures 2c and S8), and linear fitting of the K‐edge positions (Figure 2d) gives average valence states of 2.20, 2.22, and 2.27 for Co/NOC‐0.8, Co/NOC‐2.0, and Co/NOC‐3.8, respectively [18, 28]. This incremental shift indicates that a higher site density renders the Co centres more electron‐deficient through local electronic coupling. The k^3^‐weighted Fourier‐transformed (FT) extended x‐ray absorption fine structure (EXAFS) spectra (Figure 2e) display a prominent peak at ∼1.4 Å corresponding to Co‐N/O scattering. The complete absence of a Co‐Co scattering signal at ∼2.2 Å confirms that the Co species remain atomically dispersed even at the highest loading. The Co‐N/O bond length contracts systematically with increasing site density, from 1.40 Å (Co/NOC‐0.8) to 1.34 Å (Co/NOC‐2.0) and 1.32 Å (Co/NOC‐3.8). The contraction is consistent with stronger electrostatic attraction between the more electron‐deficient Co nucleus and the surrounding N/O ligands, together with a smaller effective ionic radius of the higher‐valent Co species, leading to a more compact coordination shell at higher site densities and underscoring the role of site density in shaping the local geometric and electronic structure [18, 44].

Wavelet‐transform (WT) EXAFS shows a maximum intensity at ∼4.4 Å^−1^, characteristic of the Co‐N/O scattering path (Figure 2f). A clear downshift to 3.1 Å^−1^ is observed relative to the CoPc reference, which we assign to incorporation of O into the first coordination shell, since the different backscattering amplitudes of O and N modulate the overall wave packet. This assignment is consistent with the quantitative EXAFS curve fitting (Figures S9–S13, Table S2) and confirms a Co‐N_2_O_2_ configuration. Comparative XAFS analysis of the unoxidized precursors (Figure S14) shows a blue shift of the absorption edge and an increase in white‐line intensity after oxidation [45, 46]. These changes indicate that, while the Co‐N_2_O_2_ coordination is retained (Table S2), the introduced vacancy defects further modulate the electronic structure and raise the Co valence state.

The presence and nature of the structural defects were then characterised to separate their influence from the site‐density effect. Raman spectra (Figure S15) show that the pristine Co samples exhibit low, nearly constant *I*_D_/*I*_G_ ratios (1.01–1.04), consistent with a highly graphitic carbon matrix that is independent of the initial Co loading [32, 47]. After oxidation, the *I*_D_/*I*_G_ ratios increase to 1.12–1.14 across all samples, indicating that selective etching of the metal sites during oxidation promotes vacancy formation. Consistently, the EPR spectra of the Co/NOC‐0.8, Co/NOC‐2.0, and Co/NOC‐3.8 samples exhibit intense signals at *g* = 1.996 (Figure S16), which are absent in their unoxidized counterparts, confirming the successful generation of vacancy defects and indicating comparable defect concentrations across different Co site densities [48, 49]. Moreover, C K‐edge XAS spectra exhibit a negative shift of approximately 0.2 eV in the σ* (C = C) peak (corresponding to the electron transition from C 1s to the unoccupied σ* state), indicating the generation of intrinsic sp^3^‐hybridised carbon defects after oxidation (Figure S17). Consistently, C 1s XPS spectra showed that the relative sp^3^‐hybridised carbon content increased from 17.5% to 19.5% in Co/NOC‐0.8 compared with its unoxidized counterpart, and a similar trend was observed for Co/NOC‐2.0 and Co/NOC‐3.8 (Figure S18). Therefore, these results confirm the emergence of sp^3^‐type carbon defects after oxidation, reflecting local rehybridization of the carbon lattice. These defects are intrinsically coupled to vacancy formation and together reshape the local coordination environment around the Co centres [7, 36]. Overall, oxidation generates a vacancy‐rich environment near the Co centres, producing a Co‐N_2_O_2_‐vd configuration (vd denotes vacancy defects accompanied by local sp^3^ rehybridisation) that forms preferentially next to Co sites and modulates their electronic structure and second‐shell coordination.

To determine the atomic configuration of the Co‐N_2_O_2_‐vd site, DFT calculations were performed from candidate precursor structures. Following the sequential oxidation process, three Co‐N_2_O_2_ isomers were first compared: trans‐like Co‐NONO (Figure S19a), cis‐like Co‐NOON (Figure S19b), and cis‐like Co‐OONN (Figure S19c). Trans‐like Co‐NONO has the lowest formation energy (*E*_f_ = 0.46 eV) and is therefore the thermodynamically preferred isomer. Vacancy defects were then introduced into the second coordination shell of this Co‐NONO motif, yielding five representative trans‐like Co‐NONO‐vd configurations (Figure S20). Among them, the model in Figure S20a, in which the Co atom sits in a reconstructed divacancy‐derived cavity, has the lowest formation energy (–1.48 eV) and is identified as the most stable local environment. We then performed quantitative EXAFS fitting of the unoxidised Co/NOC‐0.8 sample with the three Co‐N_2_O_2_ isomer models above. The trans‐like Co‐NONO configuration gives the best fit to the experimental data (lowest R‐factor, 0.007). For the oxidised catalyst, the corresponding trans‐like Co‐NONO‐vd model gives the best fit among all candidate structures (Figure S20, Table S3). These results support the proposed trans‐like Co‐NONO‐vd configuration. The Raman (Figure S15) and C 1s XPS (Figure S18) spectra are nearly identical across the series, showing that the carbon support configuration is essentially the same at all metal loadings. The combined evidence indicates that all Co/NOC samples retain the trans‐like Co‐NONO‐vd geometry identified by DFT.

### 2.2 Assessing the Practical Hydrogen Peroxide Output in Electrocatalytic Systems

The electrocatalytic performance of the Co SACs for H_2_O_2_ production was first evaluated by rotating ring‐disk electrode (RRDE) measurements in 0.1 M Na_2_SO_4_ [50]. Under O_2_‐saturated conditions, all samples display a plateau‐like limiting current at –0.89 V (Figure 3a), which provides a benchmark for the total ORR activity [51]. The characteristic half‐wave potentials, centred near –0.29 V, provide a kinetic reference for the selective formation of H_2_O_2_. The disk current density at –0.89 V varies nonlinearly with Co loading (Figure 3b), increasing from –2.2 mA·cm^−2^ on the Co‐free NOC to –2.8 mA·cm^−2^ on Co/NOC‐3.8 as the Co content increases from 0 to 3.83 wt.%, indicating that a higher site density scales up the total oxygen‐reduction flux at the electrode surface.

**FIGURE 3:**
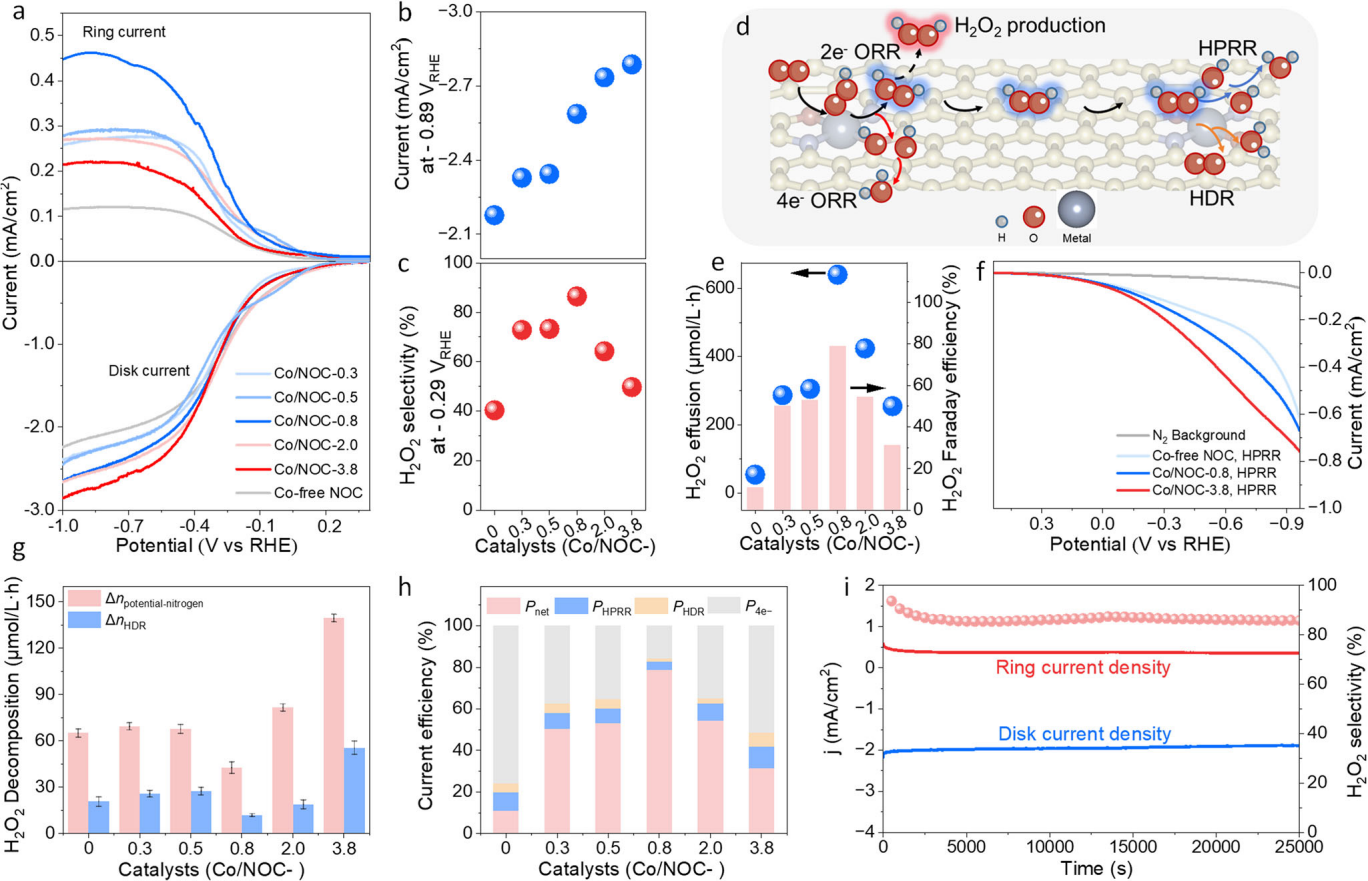
(a) LSV curves and corresponding H_2_O_2_ detection currents recorded at the ring electrode in O_2_‐saturated 0.1 M Na_2_SO_4_. (b) Current densities of various Co SACs measured at –0.89 V versus RHE. (c) Corresponding H_2_O_2_ selectivity of Co SACs measured at –0.29 V. (d) Schematic illustration of the coupled ORR, HPRR, and HDR processes occurring within the local microenvironment of adjacent Co sites, which jointly determine the practical H_2_O_2_ production. (e) H_2_O_2_ effusion rates and corresponding Faradaic efficiencies of various Co SACs. (f) ORR polarization curves in O_2_‐saturated 0.1 M Na_2_SO_4_ and HPRR polarization curves in N_2_‐saturated 0.1 M Na_2_SO_4_ containing 10 mM H_2_O_2_. (g) H_2_O_2_ decomposition in N_2_‐saturated Na_2_SO_4_ was evaluated both under an applied potential (potential‐nitrogen) and in the absence of potential (HDR) with added H_2_O_2_. The error bars represent the standard deviation of the three parallel test data. (h) Semi‐quantitative deconvolution of the current efficiency (%) into the relative contributions of net H_2_O_2_ accumulation (*P*_net_), HPRR consumption (*P*_HPRR_), HDR consumption (*P*_HDR_), and the residual contribution assigned to direct water formation through the 4e^−^‐ORR pathway ($P_{4e^{-}}$) for Co‐free NOC and Co SACs with different Co loadings. All contributions were normalised to the theoretical H_2_O_2_ output under the same electron input, which was defined as 100%. (i) Chronoamperometric response of the Co/NOC‐0.8 for stability test evaluation measured RRDE at 1600 rpm in O_2_‐saturated 0.1 M Na_2_SO_4_ for 25 000 s (applied potential: –0.5 V vs. RHE).

The H_2_O_2_ selectivity, calculated from the ring/disk current ratio (Supporting Information), shows a marked divergence in catalytic quality (Figure S21). Although high‐loading samples deliver larger total currents, their selectivity drops sharply. A clear volcano‐type relationship between selectivity and site density emerges (Figure 3c): Co/NOC‐0.8 reaches a selectivity of 86.5%, whereas the high‐density samples (e.g., Co/NOC‐3.8) shift sharply toward the unwanted 4e^−^‐ORR pathway. To further establish the site‐density dependence, Co SACs with lower Co loadings were synthesised following the identical polymerisation‐carbonisation procedure, with only the amount of Co^2+^ precursor adjusted. The resulting catalysts, denoted as Co/NOC‐0.3 and Co/NOC‐0.5, were confirmed by STEM and ICP‐MS characterisation to obtain reduced Co site densities while maintaining comparable structural characteristics to those of the previously characterised Co/NOC series (Figures S22 and S23, Table S1). Co/NOC‐0.3 and Co/NOC‐0.5 exhibited similar ORR activity (both approximately –2.3 mA·cm^−2^) and comparable H_2_O_2_ selectivity (72.9% and 73.3%, respectively), both significantly lower than that of Co/NOC‐0.8. These results suggest that at lower Co site densities, inter‐site interactions remain limited because of the increased spatial separation between neighbouring Co sites, and the observed catalytic behaviour is therefore primarily governed by the intrinsic ORR behaviour of isolated Co centres (Figure 3a–c).

To trace the origin of the density‐dependent performance, we shifted from intrinsic activity to practical H_2_O_2_ yield, necessitating a re‐examination of the limitations of conventional electrochemical H_2_O_2_ detection. Standard RRDE measurements are widely used to quantify the intrinsic 2e^−^‐ORR selectivity by eliminating mass‐transport interferences. However, this idealised configuration is inherently incapable of capturing the in‐situ H_2_O_2_ consumption that occurs under real‐world, mass‐transfer‐limited conditions. As shown in Figure S24a, escalating the rotation speeds from 700 to 2200 rpm led to a synchronised increase in both the oxygen reduction and H_2_O_2_ decomposition currents on Co/NOC‐3.8 (which exhibited the highest ORR activity among the Co/NOC series), confirming that enhanced convective mass transport effectively accelerates both reaction rates. However, the measured H_2_O_2_ selectivity exhibited an insensitivity to the rotation speeds, fluctuating only slightly from 50.1% to 55.9% at –0.29 V (Figure S24b). This independence demonstrates that even at a low rotation speed of 700 rpm, the forced convection in the RRDE setup is sufficient to achieve near‐instantaneous spatial separation of the peroxide product from the disk to the ring. In contrast, the practical H_2_O_2_ yield on a stationary plate electrode is governed by much more stringent mass‐transfer and diffusion limitations. Under these real‐world conditions, the as‐formed H_2_O_2_ has a longer residence time in the active‐site microenvironment and is more susceptible to side reactions, particularly HPRR and HDR [1, 5, 52, 53]. HPRR and HDR proceed through fundamentally different peroxide‐consumption mechanisms. HPRR involves electrochemical reduction of adsorbed H_2_O_2_ via electron/proton transfer, whereas HDR proceeds through direct H_2_O_2_ adsorption and surface‐mediated disproportionation at Co sites without external electron input, resembling a catalytic decomposition process. Both pathways occur in parallel during electrocatalysis, whereas only HDR operates in the open‐circuit catalytic system. The practical H_2_O_2_ yield is therefore not simply a measure of intrinsic generation efficiency; it is the net outcome of a kinetic competition between initial electrochemical generation and subsequent microenvironmental decomposition through the reactions above (Figure 3d) [54, 55].

To evaluate the practical H_2_O_2_ yield, bulk electrosynthesis was performed in an H‐cell equipped with a proton exchange membrane (PEM) (Figure S25). The Co‐free NOC shows a negligible H_2_O_2_ effusion rate (54.3 µmol·L^−1^·h^−1^) and a low Faradaic efficiency (FE, 10.9%) (Figure 3e), consistent with the poor intrinsic selectivity of the metal‐free carbon substrate. Introducing single Co sites produces a marked jump in performance: Co/NOC‐0.8 reaches the highest practical output (640.3 µmol·L^−1^·h^−1^) with an FE of 78.7% and a substantially higher effusion rate. However, Co/NOC‐0.3 and Co/NOC‐0.5 exhibited significantly lower H_2_O_2_ effusion rates (286.7 and 305.2 µmol·L^−1^·h^−1^, respectively) and lower Faradaic efficiencies (50.3% and 52.9%) than Co/NOC‐0.8. These results further suggest that excessively low Co site densities are insufficient to achieve efficient practical H_2_O_2_ production, as the catalytic behaviour remains primarily governed by the intrinsic properties of isolated Co centres, with inter‐site interaction playing a negligible role. By contrast, an intermediate site density provides an adequate active‐site population while maintaining controlled inter‐site coupling, thereby delivering superior practical H_2_O_2_ production. The H_2_O_2_ output then declines sharply as the Co loading exceeds 0.84 wt.%. This decay supports the microenvironmental‐trap picture: as the inter‐site distance shrinks, stronger electronic interactions develop between adjacent metal sites, making the as‐formed H_2_O_2_ more susceptible to capture by surrounding high‐density Co sites and to subsequent decomposition. The net H_2_O_2_ spillover efficiency is therefore maximised only at an optimal low site density that balances primary generation against the suppression of secondary decomposition.

To test the hypothesis that an optimised site density suppresses parasitic consumption, we decoupled electrochemical generation (ORR) from the competing consumption pathways (HPRR and HDR) using independent kinetic measurements [56]. Specifically, HDR was quantified under open‐circuit conditions with externally added H_2_O_2_, where electrochemical reduction cannot occur and the measured peroxide consumption exclusively originates from catalytic disproportionation. In contrast, HPRR was evaluated under an N_2_ atmosphere with externally supplied H_2_O_2_ and applied potential, where both current responses in LSV and H_2_O_2_ consumption in a plate‐electrode system were jointly considered to cross‐validate the contribution of electrochemical peroxide reduction and improve the reliability of pathway discrimination. In the LSV curves recorded under N_2_ with added H_2_O_2_ (Figure 3f), the reduction current density at –0.89 V increases from –0.65 mA·cm^−2^ on the Co‐free NOC to –0.77 mA·cm^−2^ on Co/NOC‐3.8. This trend confirms that Co single‐atom centres serve as active sites for the electrochemical H_2_O_2_ reduction. Based on these decoupled electrocatalytic and heterogeneous catalytic platforms (Notes S1 and S2), the kinetic rates of HPRR and HDR were precisely quantified by monitoring H_2_O_2_ consumption in both N_2_‐saturated electrocatalytic and heterogeneous catalytic systems with externally added H_2_O_2_, enabling us to determine their individual contributions to the overall peroxide consumption during the reaction (Table S4). As the Co loading increased from 0.27 to 0.84 wt.%, the total H_2_O_2_ consumption (Δ*n*_potential‐nitrogen_) decreased from 69.3 to 42.6 µmol·L^−1^·h^−1^ and then rose sharply to 139.4 µmol·L^−1^·h^−1^ as the loading was further scaled up to 3.83 wt.% (Figure 3g). Similarly, the purely chemical HDR tracked a matching trajectory, peaking at 3.83 wt.% (55.5 µmol·L^−1^·h^−1^). Consequently, Co/NOC‐0.8 stands out as the optimal site density, exhibiting substantially lower consumption rates (HPRR 30.8 µmol·L^−1^·h^−1^, HDR 11.8 µmol·L^−1^·h^−1^) than its low‐density (Co/NOC‐0.3: HPRR 43.6 µmol·L^−1^·h^−1^, HDR 25.8 µmol·L^−1^·h^−1^) and high‐density counterparts (Co/NOC‐3.8: HPRR 83.9 µmol·L^−1^·h^−1^, HDR 55.5 µmol·L^−1^·h^−1^). These results convincingly demonstrate that adjusting the Co site to its optimum density (Co/NOC‐0.8) creates an ideal geometric equilibrium, which not only suppresses the parasitic HPRR and HDR pathways by approximately 63% and 79% relative to the high‐density configuration (Co/NOC‐3.8), but also mitigates these competing reactions by 29% and 54% compared to the low‐density control (Co/NOC‐0.3).

Building on these kinetic results, we developed an H_2_O_2_‐equivalent pathway deconvolution model that partitions the current efficiency into four distinct fates (Note S2, Tables S4 and S5): net H_2_O_2_ (*P*_net_), HPRR loss (*P*_HPRR_), HDR loss (*P*_HDR_), and direct water formation through the 4e^−^‐ORR pathway ($P_{4e^{-}}$). In this scheme, the theoretical H_2_O_2_ output of a given catalyst under a given operating current and reaction time, assuming 100% Faradaic efficiency toward the 2e^−^‐ORR pathway, serves as the common reference baseline. The applicability of this model is limited to operating conditions where the measured current is predominantly associated with O_2_ reduction, rather than competing electrochemical processes such as hydrogen evolution, capacitive charging, catalyst redox transitions, or electrolyte reduction. Under this condition, the input charge can be reasonably converted into an H_2_O_2_‐equivalent baseline and subsequently partitioned into net H_2_O_2_ accumulation, HPRR loss, HDR loss, and direct 4e^−^‐ORR ‐derived water formation. The $P_{4e^{-}}$ fraction represents the portion of the input current intrinsically diverted to the 4e^−^‐ORR pathway during the initial O_2_ reduction step, leading to direct H_2_O formation. This step is governed by inter‐site electronic interactions and determines the intrinsic ORR selectivity. The remaining fraction corresponds to the subsequent fate of the electrogenerated H_2_O_2_ produced via the 2e^−^‐ORR pathway, partitioned between *P*_net_, *P*_HPRR_ and *P*_HDR_. Figure 3h provides further insight into the density‐dependent performance. For intrinsic H_2_O_2_ generation, the total primary H_2_O_2_ production (sum of *P*_net_, *P*_HPRR_, and *P*_HDR_) jumps stepwise on the introduction of isolated Co sites. The primary H_2_O_2_ current efficiency of Co/NOC‐0.8 (84.0%) reaches 3.5 and 1.3 times higher than those of the Co‐free NOC (24.0%) and low‐density Co/NOC‐0.3 (62.4%) counterparts, respectively. This selectivity gain reflects a mechanistic transition from a dominant 4e^−^‐ORR pathway on the carbon substrate to the 2e^−^‐ORR pathway on the Co centres. Excessive site density (Co/NOC‐2.0 and Co/NOC‐3.8) compromises this intrinsic selectivity and shifts the reaction toward unwanted pathways. For the net retention of H_2_O_2_, the optimised site isolation in Co/NOC‐0.8 limits secondary consumption of the as‐formed H_2_O_2_, with its total parasitic losses (*P*_HPRR_ + *P*_HDR_) suppressed to a negligible value (5.3%), which sits well below those of low‐density (Co/NOC‐0.3, 12.2%), high‐density (Co/NOC‐3.8, 17.1%) and even the Co‐free (Co‐free NOC, 13.1%) counterparts. These results quantitatively unveil a density‐control shift from isolated‐site‐dominated catalysis at lower Co site densities to interaction‐regulated behaviour at elevated site densities. An intermediate site density (Co/NOC‐0.8) represents the optimal configuration, providing a sufficient population of active sites while maintaining controlled inter‐site coupling, thereby maximising H_2_O_2_ generation and suppressing its subsequent decomposition. Moreover, Co/NOC‐0.8 maintains a nearly constant current density and an H_2_O_2_ selectivity above 86.5% over 25 000 s in neutral electrolyte (Figure 3i), indicating durable operation with sustained 2e^−^‐ORR selectivity [28]. Overall, engineering such an intermediate site density provides site proximity that balances intrinsic 2e^−^‐ORR selectivity with maximal product spillover, yielding long‐term catalytic stability and high Faradaic efficiency.

### 2.3 Mechanism Insights of the Co Active Site

To probe the origin of the catalytic activity, SCN^−^ poisoning experiments were first performed on catalysts with different site densities. On addition of SCN^−^, the ring current (which tracks H_2_O_2_ production) dropped to the baseline value of the metal‐free NOC while the disk current remained stable (Figure S26). This result confirms that the Co‐N_2_O_2_‐vd moieties are the sole active centres for H_2_O_2_ electrosynthesis. The poisoning‐induced change in the Co coordination environment also drives a mechanistic shift from the 2e^−^‐ORR to the 4e^−^‐ORR pathway [52]. The mere presence of Co single atoms is not, however, sufficient to guarantee high 2e^−^‐ORR selectivity. Control experiments on the unoxidised samples show that, regardless of Co loading, all unoxidised catalysts favour the 4e^−^‐ORR pathway, with limiting current densities above 3.0 mA cm^−2^ and H_2_O_2_ selectivity below 40% (Figure S27). The metal‐free NOC alone also shows poor intrinsic 2e^−^‐ORR selectivity despite its defect‐rich nature (Figure 3c). This three‐way contrast indicates that the vacancy defects are not isolated active sites in their own right; rather, they form a necessary microenvironment in the second coordination sphere that electronically modulates the Co centres, steering the initial oxygen activation toward the 2e^−^‐ORR pathway while raising the thermodynamic barrier to H_2_O_2_ decomposition.

To track the molecular evolution of this synergistic electrocatalysis, in‐situ attenuated total reflectance Fourier‐transform infrared (ATR‐FTIR) spectroscopy was carried out during the ORR (Figure S28). Four characteristic vibrational bands were identified: O_2_* (∼1410 cm^−1^), H_2_O_2_* (∼1376 cm^−1^), OOH* (∼1176 cm^−1^), and O_2_^−^* (∼1110 cm^−1^) [57, 58]. For the Co‐free NOC (Figure S29), the O_2_* band is absent, and the other intermediate signals show no appreciable potential dependence. This indicates that the metal‐free carbon substrate lacks sufficient active sites for effective oxygen adsorption. Even as the overpotential is increased, the reaction cannot overcome the initial mass‐transfer and adsorption barriers, giving sluggish 2e^−^‐ORR kinetics. This confirms that introducing Co sites is a prerequisite for oxygen binding and activation. By contrast, Co/NOC‐0.8 shows pronounced and synchronised growth of the O_2_*, OOH*, and O_2_^−^* bands as the potential is increased (Figure 4a). No H_2_O_2_* accumulation band is detected at ∼1376 cm^−1^, indicating that O_2_ is rapidly reduced to H_2_O_2_ on the isolated Co sites, and the H_2_O_2_ then diffuses promptly into the bulk electrolyte rather than residing on the catalyst surface [59]. This consecutive generation and rapid release contribute to the high Faradaic efficiency and net yield of Co/NOC‐0.8. The spectroscopic profile of Co/NOC‐3.8, with a higher cobalt loading, is different (Figure S29). Alongside strong O_2_*, OOH*, and O_2_^−^* bands, a prominent H_2_O_2_* accumulation peak appears at ∼1376 cm^−1^, indicating that the as‐formed H_2_O_2_ is trapped and enriched at the catalyst‐electrolyte interface, consistent with the DFT‐calculated H_2_O_2_ adsorption energetics presented below [60]. The longer surface residence time then drives further decomposition of H_2_O_2_ via HPRR and HDR on the adjacent Co sites [34].

**FIGURE 4:**
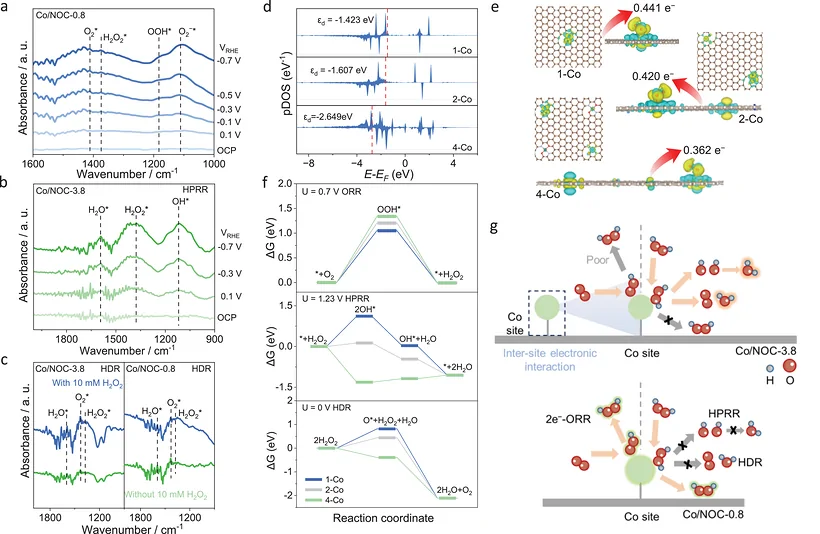
In‐ situ ATR‐FTIR spectra of (a) Co/NOC‐0.8 in O_2_‐saturated 0.1 M Na_2_SO_4_ over a potential range from 0.1 to –0.7 V, (b) Co/NOC‐3.8 in N_2_‐saturated 0.1 M Na_2_SO_4_ containing 10 mM of H_2_O_2_ at different potential, (c) Co/NOC‐3.8 and Co/NOC‐0.8 in 0.1 M Na_2_SO_4_ containing 10 mM H_2_O_2_. (d) Projected density of state (pDOS) for the Co 3d orbital for 1‐Co, 2‐Co, and 4‐Co. (e) Differential charge distribution on 1‐Co, 2‐Co and 4‐Co with adsorption of OOH* (Top and side view). (f) The Gibbs free energy profile for the 2e^−^‐ORR at U = 0.7 V versus RHE, HPRR at U = 1.23 V and HDR on different models. (g) Schematic illustration of the density‐dependent inter‐site electronic interaction between Co centres in high‐density Co/NOC‐3.8 (upper) and low‐density Co/NOC‐0.8 (lower), governing the net H_2_O_2_ production through the coupled pathways of 2e^−^‐ORR, HPRR, and HDR.

To probe the parasitic consumption of H_2_O_2_, the real‐time evolution of adsorbed intermediates was further monitored in an N_2_‐saturated electrolyte containing H_2_O_2_. Compared with the O_2_‐saturated ORR system, two additional vibrational bands appear at ∼1592 cm^−1^ and ∼1118 cm^−1^ (Figure 4b), assigned to the bending mode of H_2_O* and the stretching mode of OH*, respectively. OH* is a key intermediate in the HPRR pathway (H_2_O_2_ → 2OH*, OH* + H^+^ + e^−^ → H_2_O) [53, 61, 62]. On the Co/NOC‐3.8 surface, both the OH* and H_2_O* bands intensify with increasingly negative potential (Figure 4b), confirming that high‐density Co sites act as sinks that decompose H_2_O_2_ to H_2_O through the HPRR pathway [62]. On the Co/NOC‐0.8 surface, by contrast, the OH* and H_2_O* signals remain negligible and invariant across the investigated potential range (Figure S30). The HDR (2H_2_O_2_ → 2H_2_O + O_2_) was likewise monitored under open‐circuit (non‐electrocatalytic) conditions on the addition of H_2_O_2_. Whereas the HPRR is mediated by OH* intermediates, the HDR shows no detectable OH* band at ∼1118 cm^−1^. Instead, a characteristic O_2_* peak appears at ∼1410 cm^−1^. HDR therefore proceeds through a heterogeneous pathway in which H_2_O_2_ disproportionates directly into H_2_O and O_2_, rather than through a proton‐coupled electron transfer driven by electrocatalysis. For Co/NOC‐3.8, prominent H_2_O_2_* and O_2_* signals appear immediately on H_2_O_2_ addition (Figure 4c), indicating high activity of the densely populated Co sites toward H_2_O_2_ decomposition. The concurrent O_2_ adsorption band provides direct evidence of H_2_O_2_ disproportionation at the dense Co‐N_2_O_2_‐vd interfaces. By contrast, Co/NOC‐0.8 shows negligible spectroscopic changes on H_2_O_2_ addition, indicating that the defect‐engineered, isolated Co sites are essentially inactive toward H_2_O_2_ decomposition and so support high product spillover efficiency (Figures 4c and S31).

### 2.4 Theoretical Understanding of the Difference of Co SACs With Various Densities

To examine how site density affects the electronic structure of the Co single atoms, a series of models was constructed by embedding different numbers of Co‐N_2_O_2_‐vd moieties in a quasi‐square graphene supercell (∼30 × 30 Å^2^), giving a direct mapping between the theoretical site population and the experimental mass loading. The Co SACs derived from the polymerisation‐pyrolysis process were assumed to be uniformly distributed within the carbon matrix, a commonly adopted approximation in DFT simulations of atomically dispersed catalysts. Under this assumption, varying numbers of Co‐N_2_O_2_‐vd active sites were incorporated into graphene supercells of comparable dimensions to construct a series of site‐density models. The theoretical Co loading (wt.%) of each model was then calculated from the atomic mass ratio of Co, N, O, and C within the supercell. By systematically adjusting the graphene supercell size and relative Co‐site positions, a structurally consistent carbon framework was maintained while maximising agreement between the theoretical Co wt.% and the experimentally determined Co contents from inductively coupled plasma mass spectrometry (ICP‐MS) [17, 18]. Accordingly, a single site corresponds to 0.99 wt.%, matching Co/NOC‐0.8 (Figure S32); two sites correspond to 2.03 wt.% (Co/NOC‐2.0, Figure S33); and four sites correspond to 3.97 wt.% (Co/NOC‐3.8, Figure S34). This quantitative correspondence was used to interpret the density‐dependent catalytic behaviour of the as‐synthesised SACs. To maintain computational feasibility while preserving the local coordination environment and site distribution, an equivalent one‐quarter section of each optimised supercell was extracted as the computational unit for subsequent electronic structure and reaction pathway calculations.

The transition from fully isolated Co sites to electronically coupled, densely populated single atoms also alter the electronic structure. Projected density of states (pDOS) analysis (Figures 4d and S35) shows that the Co d‐band centre shifts markedly downward as the site density increases, from –1.423 eV on 1‐Co to –2.649 eV on 4‐Co, consistent with a weaker metal‐adsorbate interaction. This electronic reconfiguration, arising from stronger orbital hybridisation between neighbouring Co sites, raises the Co oxidation state, as also seen in the XANES data (Figure 2d). The spin magnetic moment also decreases progressively from 1.00 to 0.72 μB as the Co loading rises, further indicating a transition from isolated Co states to electronically interacting states among neighbouring Co sites [12, 19, 49]. These coupled, electron‐deficient Co centres show a reduced ability to back‐donate electrons into the antibonding π* orbitals of the O_2_ and OOH* intermediates. A moderate decrease in back‐donation generally helps to suppress O‐O bond cleavage, but the larger d‐band downshift in the high‐density 4‐Co configuration weakens the interaction with OOH* beyond the optimal range, hindering its formation and suppressing the 2e^−^‐ORR pathway. The higher limiting current observed for Co/NOC‐3.8 (4‐Co), therefore, reflects a higher density of accessible Co active sites and a higher overall O_2_ reduction turnover (Figure 3b), rather than an intrinsically improved 2e^−^‐ORR capability. The apparent activity (current density) is therefore dominated by site density and mass transport, while the reaction selectivity is governed by the stabilisation of key intermediates such as OOH*.

Charge‐density‐difference and Bader‐charge analyses (Figure 4e) provide a visual and quantitative picture of this electronic control over the key OOH* intermediate [50]. In the isolated 1‐Co model, the higher d‐band centre gives a favourable energy alignment with the O π* orbitals. This drives a concentrated, efficient electron transfer (0.441 e^−^) within the Co‐O bonding region. The charge‐density difference reveals substantial orbital overlap that stabilises the peroxide species and promotes the 2e^−^‐ORR pathway. In the high‐density 4‐Co model, the large d‐band downshift and inter‐site competition for the carbon electron pool reduce the site's ability to selectively stabilise the intermediate. The resulting electronic environment gives a more diffuse charge distribution and a markedly reduced net electron transfer to OOH* (0.362 e^−^). This weaker interaction, together with the inter‐site electronic coupling arising from the close spacing of the Co sites, provides insufficient stabilisation of the OOH* intermediate, hindering the 2e^−^‐ORR pathway and promoting competing processes involving H_2_O_2_ re‐adsorption and subsequent consumption [7, 26, 33, 63].

To build a thermodynamic picture of the density‐dependent performance, the Gibbs free‐energy profiles for H_2_O_2_ generation and for its secondary consumption were calculated. At a thermodynamic equilibrium potential of 0.7 V, the protonation of O_2_ to OOH* is the rate‐determining step (RDS) for all investigated models (Figure 4f) [64]. The free‐energy barrier (Δ*G*_OOH*_) decreases markedly from 1.34 eV on the 4‐Co model to 1.05 eV on 1‐Co. This trend indicates that an isolated Co site lowers the activation energy for H_2_O_2_ formation. The Gibbs free‐energy profiles for HPRR and HDR were also calculated at their respective thermodynamic equilibrium potentials of 1.23 and 0 V. The 4‐Co model shows the most favourable H_2_O_2_ adsorption, with an adsorption energy of −1.14 eV, in clear contrast to the unfavourable values (−0.53 and −0.79 eV) for 1‐Co and 2‐Co, respectively (Figure S36). This trend is consistent with the in‐situ ATR‐FTIR results, which show pronounced H_2_O_2_ accumulation on high‐density Co sites and thus strong surface retention (Figures S30 and S31). As shown in Figure 4f, the conversion of H_2_O_2_ to OH* remains highly unfavourable on 1‐Co, with a steep energy barrier of 1.12 eV [2, 65, 66]. On 4‐Co, by contrast, this step becomes energetically downhill, indicating a spontaneous tendency for H_2_O_2_ to be reduced to H_2_O. For the HDR pathway, 1‐Co requires a substantial energy barrier (0.81 eV), whereas 4‐Co shows a negative overpotential (−0.39 eV). These results confirm that high site density promotes the spontaneous decomposition of H_2_O_2_.

Taken together, these findings support a Co density‐dependent mechanism for H_2_O_2_ electrosynthesis over Co‐N_2_O_2_‐vd SACs. As shown in Figure 4g, the atomic Co sites serve as the primary active centres and have a high intrinsic affinity for O_2_. The reaction begins with O_2_ adsorption, followed by protonation to form the key intermediate OOH*. At low site densities (Co/NOC‐0.8), the Co centres remain site‐isolated and free from electronic interference from neighbouring metal sites. The localised electronic environment enables optimal stabilisation of the OOH* intermediate and avoids the insufficient binding seen at high‐density, electron‐deficient Co sites. Once H_2_O_2_ is formed, the absence of adjacent Co sites that would otherwise act as H_2_O_2_‐trapping centres with high HPRR and HDR activity allows rapid diffusion (spillover) from the catalyst surface into the bulk electrolyte, thereby suppressing in‐situ consumption and giving high Faradaic efficiency and selectivity. In comparison, as the single‐atom density increases (Co/NOC‐3.8), the close spacing of adjacent Co sites produces strong electronic coupling. The overlapping electronic fields and d‐band reconfiguration weaken the stabilisation of the OOH* intermediate beyond the optimal regime, suppressing the 2e^−^‐ORR pathway and allowing the dense Co sites to act as reactive traps that promote H_2_O_2_ re‐adsorption and its consumption through thermodynamically favourable HPRR and HDR processes.

### 2.5 Electrified Membrane for Sustainable Environmental Applications

As shown in Figure 5a, a Co SAC/CNT‐EM cathode (Figure 5b) is placed upstream for in‐situ electrosynthesis of H_2_O_2_, and a FeOCl/CNT‐M layer (Figure 5e) is integrated downstream as the activation counterpart [67, 68, 69, 70, 71]. FeOCl/CNT‐M was selected as the activation layer because of its high intrinsic activity towards heterogeneous H_2_O_2_ activation and efficient Fe(III)/Fe(II) redox cycling under flow‐through conditions. In addition, its membrane‐compatible architecture enables localised radical generation while avoiding continuous dissolved iron dosing and associated secondary contamination. This spatial arrangement enables an efficient generation‐activation cascade. As contaminated water permeates the module, H_2_O_2_ produced at the upstream cathode is immediately captured and converted into hydroxyl radicals by the downstream FeOCl layer. On‐demand radical generation minimises the loss of active species and maximises the oxidation flux within the confined flow‐through channels.

**FIGURE 5:**
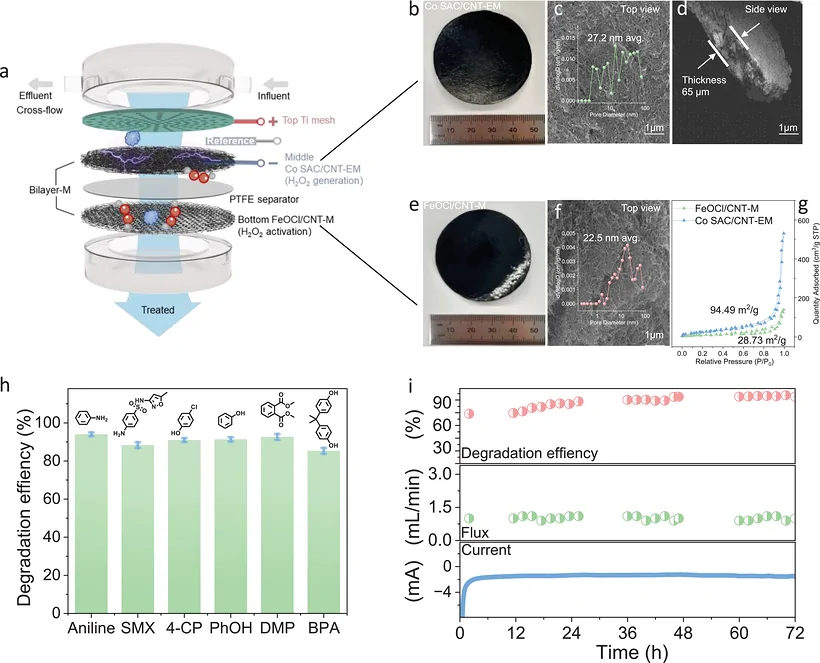
(a) Schematic illustration of the crossflow single‐pass electrified membrane filtration system. The self‐fabricated membrane (black) with an effective area of 19.6 cm^2^ and the Ti mesh (green) serve as the cathode and anode, respectively. The FeOCl/CNT‐M at the bottom serves as the in‐situ generated H_2_O_2_ activator. (b) Photographs and SEM images of the (c) top and (d) cross‐sectional views of the Co SAC/CNT‐EM (middle). (e) Photographs and (f) top‐view SEM images of FeOCl/CNT‐M (bottom), and (g) N_2_ sorption isotherms of Co SAC/CNT‐EM and FeOCl/CNT‐M. Insets in the SEM images show the pore size distributions and average pore diameters. (h) Removal performance of the bilayer electrified membrane filtration system toward different contaminants (conditions: [Aniline] = [SMX] = [4‐CP] = [PhOH] = [DMP] = [BPA] = 1 mg·L^−1^, O_2_‐saturated, neutral pH, crossflow rate of 200 mL·min^−1^, recirculating feed solution containing 0.1 M Na_2_SO_4_ and pollutants, room temperature). (i) Long‐term stability of water flux, current, and removal efficiency for 1 mg·L^−1^ aniline over 72 h of continuous operation.

A closer examination of the bilayer assembly reveals the structural basis for its high catalytic efficiency. The Co SAC/CNT‐EM has an interwoven porous network with an average pore size of ∼27 nm (Figure 5c) and a high specific surface area of 94.49 m^2^·g^−1^ (Figure 5g). Cross‐sectional SEM imaging (Figure 5d) shows a well‐defined lamellar architecture for the Co SAC/CNT‐EM with a thickness of about 65 µm. This hierarchical porosity is critical for flow‐through operation, as it ensures sufficient exposure of the Co single sites and promotes convection‐enhanced mass transport [69]. The compact, tightly integrated FeOCl membrane in the downstream layer of the bilayer (Figure 5e,f) reduces the diffusion barrier and enables rapid, sufficient contact between the short‐lived radicals and pollutant molecules during the rapid permeate flow. The operational performance of the integrated module was evaluated against a diverse library of organic micropollutants. The system achieved high removal efficiencies for a range of pharmaceutical (e.g., sulfamethoxazole) and industrial (e.g., aniline, phenol, bisphenol A) contaminants under neutral conditions (Figure 5h). All target pollutants were effectively degraded in single‐pass mode with a hydraulic residence time of only ∼8 s. This rapid treatment shows that the combination of an optimised Co site density and a modular cascade design can overcome key limitations of conventional Fenton‐like processes, namely, sluggish mass transfer, poor utilisation of short‐lived reactive species, and the need for extended reaction times. To assess operational stability, a long‐term chronopotentiometry test was performed under continuous crossflow conditions using a 1 mg·L^−1^ aniline solution (Figure 5i). The cathodic current and permeate flux remained stable for 72 h of continuous operation, and the single‐pass aniline degradation efficiency remained at about 90% for most of the testing period. ICP‐MS analysis after long‐term operation detected only trace metal leaching (Co: 16.02 µg·L^−1^; Fe: 15.19 µg·L^−1^; Table S6), indicating good structural stability of the electrified membrane system and suggesting a low risk of secondary contamination during operation. Furthermore, the flow‐through electrified membrane system exhibited a low energy consumption per order (EC_EO_) of 0.054 kWh·m^−3^·order^−1^, outperforming representative advanced electro‐Fenton systems reported in recent years, including both flow‐through reactors and conventional single‐cell electrolysis configurations (0.102–4.20 kWh·m^−3^·order^−1^, Table S7). Overall, the Co/NOC‐0.8‐driven electrified membrane system enables efficient pollutant removal with stable long‐term operation and low energy demand, highlighting its strong potential for practical implementation and future scale‐up in continuous micropollutant remediation.

## 3 Conclusion

In summary, we have identified Co site density as a key regulator that balances H_2_O_2_ generation against its in‐situ decomposition over defect‐rich Co‐N_2_O_2_ SACs. The atomic Co centre is the primary active site for oxygen activation, and site isolation is the decisive factor in preserving H_2_O_2_ as the target product. The low‐loading Co/NOC‐0.8 catalyst delivers an optimal Faradaic efficiency of 78.7%, a 2.5‐fold improvement over the high‐density counterpart Co/NOC‐3.8. Kinetic analysis shows that optimised site isolation suppresses HPRR and HDR by 63% and 79%, respectively, removing the H_2_O_2_ over‐decomposition that limits densely populated SACs. Mechanistic analysis indicates that a reduced site density limits orbital hybridisation and electron delocalisation between adjacent Co sites, preventing d‐band broadening and maintaining a relatively high d‐band centre. This electronic structure lowers the energy barrier for the O_2_ → OOH* step while thermodynamically suppressing dissociation of H_2_O_2_ into OH* and O*, giving high‐yield H_2_O_2_ production. To demonstrate the practical potential, a continuous‐flow electrocatalytic membrane reactor was built using Co/NOC‐0.8 and FeOCl for in‐situ H_2_O_2_ generation and activation. The integrated system delivered high‐efficiency wastewater treatment, achieving > 90% single‐pass removal of aniline and other micropollutants with stable operation over 72 h. This work establishes a link between inter‐site electronic interactions and catalytic performance in densely packed SACs and points to the potential of in‐situ electrochemical H_2_O_2_ for practical environmental remediation.

## Author Contributions

**Junwen Chen**: conceptualization, data curation, investigation, formal analysis, writing – original draft. **Hongyu Zhou**: methodology, formal analysis. **Qiming Zhang**: investigation. **Shuang Zhong**: investigation, methodology, validation. **Wei Ren**: writing – review and editing, supervision, formal analysis. **Lei Shi**: investigation. **Li Gao**: methodology. **Shaobin Wang**: writing – review and editing, formal analysis. **Xiaoguang Duan**: conceptualization, investigation, project administration, resources, supervision, writing – review and editing, funding acquisition.

## Conflicts of Interest

The authors declare no conflicts of interest.

## Supporting information




**Supporting File**: anie73778‐sup‐0001‐SuppMat.docx.

## Data Availability

The data that support the finding of this study will be made available upon request from authors.
