# Supplementary material for: Decouple H2O2 Electrosynthesis From Microenvironmental Decomposition via Atomic Site Density Engineering
Source: Angew Chem Int Ed Engl. 2026 Aug 5;65(40):e8125244. doi: 10.1002/anie.8125244 (PMC13618308; doi:10.1002/anie.8125244)
Supplement: Supplementary file 1 [file ANIE-65-e8125244-s001.docx]

*Supporting Information*

**Decouple H_2_O_2_ Electrosynthesis from Microenvironmental Decomposition via Atomic Site Density Engineering**

Junwen Chen^a^, Hongyu Zhou^a^, Qiming Zhang ^a^, Shuang Zhong^a^, Wei Ren^a,^*, Lei Shi^b^, Li Gao^c^, Shaobin Wang^a^, Xiaoguang Duan^a,^*

^a^ School of Chemical Engineering, Adelaide University, Adelaide, SA5005, Australia

^b^ College of Materials Science and Engineering, Nanjing Forestry University, Nanjing 210037, China

^c^ School of Science, STEM College, RMIT University, Melbourne, VIC, 3000, Australia

** Corresponding author*. E-mail:	wei.ren@adelaide.edu.au (W. Ren),

[xiaoguang.duan@adelaide.edu.au](mailto:xiaoguang.duan@adelaide.edu.au) (X. Duan).

## Table of Contents

This file includes:

1. Experimental Procedures
2. Supplementary Notes S1‒S3
3. Supplementary Figures S1‒S40
4. Supplementary Tables S1‒S7
5. References
6. Author Contributions

## 1. Experimental Procedures

**Synthesis of Co/NOC catalysts**

The Co/NOC-0.8 samples were synthesized following a previously reported procedure with slight modification. Briefly, melamine (2 g), cyanuric acid (2 g), L-alanine (2 g), and phytic acid solution (50 wt%, 200 μL) were dispersed in 100 mL of deionized water and vigorously stirred at 100 °C to form a homogeneous polymeric precursor. Subsequently, 36 mg of Co(NO_3_)_2_·6H_2_O was slowly added under continuous stirring for 12 hours to yield a light pink slurry, which was rapidly frozen in liquid nitrogen and freeze-dried for three days. The obtained solid was then pyrolyzed at 750 °C for 2 h under an Ar atmosphere to afford a black powder. The resulting material was treated in 1 M HCl at 60 °C for 24 h under stirring, washed with deionized water until neutral, and vacuum-dried to obtain Co/NOC-0.8-unoxidised. Finally, 10 mg of Co/NOC-0.8-unoxidised powder was dispersed in 10 mL of nitric acid and heated at 80 °C for 0.5 h under stirring, followed by dilution, washing, filtration, and freeze-drying to produce the final Co/NOC-0.8 sample. The syntheses of Co/NOC-2.0 and Co/NOC-3.8 followed identical procedures, employing 81 mg and 135 mg of Co(NO_3_)_2_·6H_2_O, respectively. In contrast, Co-free NOC was synthesized using the same protocol without cobalt salt addition.

**Characterization and analysis methods**

Inductively coupled plasma mass spectrometry (ICP-MS; Agilent 8900x QQQ) quantified metal loading on catalysts. Catalyst and membrane morphologies were characterized by field emission scanning electron microscopy (SEM; JEOL JSM-7500F) and transmission electron microscopy (TEM; FEI Tecnai G2 F20 S-TWIN). X-ray powder diffraction (XRD) patterns were acquired using a Rigaku MiniFlex 600 diffractometer. Elemental mapping via high-angle annular dark-field scanning TEM (HAADF-STEM) with energy-dispersive X-ray spectroscopy (EDX) was performed on an FEI Titan Themis 80-200. Surface chemistry was analyzed by: X-ray photoelectron spectroscopy (XPS; Thermo Fisher ESCALAB 250Xi), Raman spectroscopy (LabRAM HR, HORIBA), Fourier transform-infrared (FTIR) spectroscopy (Nicolet 6700, Thermo Fisher Scientific). Electron paramagnetic resonance (EPR; Bruker-5000 spectrometer) characterized sample defects. Specific surface areas of catalysts and membranes were determined by Brunauer-Emmet-Teller (BET) analysis of N_2_ adsorption-desorption isotherms (V-Sorb 2800 P analyzer). Hydrogen peroxide concentrations were quantified by UV-vis spectrophotometry (Shimadzu UV-1800), while target organics were measured via HPLC (C18 column). X-ray absorption spectroscopy (XAS) measurements conducted at the Australian Synchrotron employed metal foils and oxides as references. Both X-ray absorption near-edge structure (XANES) and extended X-ray absorption fine structure (EXAFS) data were processed using QANT and IFEFFIT’s ATHENA/ARTEMIS modules.

**Electrochemical measurements**

LSV testing was conducted using a rotating ring-disk electrode (RRDE) assembly, consisting of a glassy-carbon disk electrode (disk area = 0.1963 cm^2^) and a Pt ring with a theoretical collection efficiency (N) of 25%. Measurements were performed on the bare RRDE in an oxygen-free electrolyte (1 M KCl + 10 mM K_3_[Fe(CN)_6_]) at various rotation speeds, with the disk electrode potential scanned from 1.1 V to 0.25 V (vs. RHE) at 10 mV s^−1^ to reduce Fe^3+^ to Fe^2+^, while the Pt ring was held at 1.1 V (vs. RHE) to oxidize Fe^2+^ to Fe^3+^. As shown in Figure S40, the collection efficiency was calculated to be 0.25, independent of rotation speed, using N = –I_ring_/I_disk_, ring current (I_ring_), disk current (I_disk_).

The disk is used for electrochemical ORR processes, with its rotation enhancing mass transfer and reaction kinetics. The Pt ring electrode detects reaction products and intermediates. The catalyst ink was prepared by dispersing 2 mg of electrocatalyst in 495 μL of 2-propanol, 495 μL of pure water, and 10 μL of nafion solution (5.0 wt.%), followed by sonication for 60 minutes. A 10 μL portion of the resulting suspension was pipetted onto the disk electrode and allowed to dry naturally. Electrochemical measurements were performed in an O_2_-saturated 0.1 M Na_2_SO_4_ solution at room temperature (25 °C). The RRDE was rotated at 1600 rpm, and the Pt ring potential was set to 1.23 V (vs. RHE) to oxidize H_2_O_2_ generated at the disk electrode. Linear sweep voltammetry (LSV) was carried out at a scan rate of 10 mV·s^−1^. The selectivity of hydrogen peroxide (H_2_O_2_ %) was calculated from the disk current (I_D_) and ring current (I_R_) using the equations below.

H_2_O_2_% = 200 × (I_R_ /N) / (| I_D_ | + I_R_ /N)

n = 4× | ID | / (| ID | + IR /N)

The Faraday efficiency was calculated based on the following,

Faradaic efficiency (%) = 2CVF/Q×100

Where C, V, F and Q are the cumulative concentration of H_2_O_2_ (mol·L^−1^), total electrolyte volume (L), Faraday constant (96,485 C·mol^−1^) and quantity of charge passed (C) respectively. Chronoamperometry at –0.5 V vs RHE (Figure 3i) assessed H_2_O_2_ electrosynthesis stability. H_2_O_2_ yield experiments utilized a standard three-electrode H-cell containing O_2_-saturated 0.1 M Na_2_SO_4_ electrolyte, with catalyst coated on hydrophilic carbon paper (loading: 1 mg cm^−2^; dimensions: 2 × 2 cm^2^).

**Qualification of H_2_O_2_**

The H_2_O_2_ concentration-absorbance calibration curve was generated through linear regression of absorbance values measured at 400 nm, corresponding to the orange complex formed between H_2_O_2_ and titanium potassium oxalate (TPO) under acidic conditions. Initially, 0.05 M TPO solution and concentrated H_2_SO_4_ were prepared. For analysis, aliquots were withdrawn from the reaction solution; 3 mL of each aliquot was mixed with 0.5 mL of 0.05 M TPO and 0.5 mL concentrated H_2_SO_4_. After vortex mixing and a 30-second reaction period, the absorbance at 400 nm was measured using UV-vis spectrophotometry to quantify H_2_O_2_ concentration.

**Details of *in situ* ATR-FTIR experiment involving electrochemical ORR, HPRR and HDR**

*In situ* ATR-FTIR measurements were performed using a Thermo-Fisher Nicolet iS20 spectrometer equipped with a liquid nitrogen-cooled HgCdTe (MCT) detector and a Pike VeeMax III ATR accessory. A germanium prism integrated into a PIKE electrochemical cell (three-electrode configuration: Ag/AgCl reference, Pt-wire counter) served as the working electrode substrate. The catalyst was deposited via airbrushing onto the prism. Spectra were acquired by averaging 64 scans at 4 cm^−1^ resolution. For the 2e^−^-ORR experiments, a 0.1 M Na_2_SO_4_ electrolyte (pH 5.73) was continuously purged with O_2_. Chronoamperometry was conducted stepwise at discrete potentials from 0.1 to –0.9 V vs. RHE, with open-circuit potential (OCP) spectra collected as baseline. For hydrogen peroxide reduction (HPRR), tests employed N_2_-saturated 0.1 M Na_2_SO_4_ containing 10 mM H_2_O_2_, scanning from 0.1 to –0.7 V vs. RHE. Hydrogen decomposition (HDR) studies used identical H_2_O_2_-containing N_2_-saturated electrolyte without applied potential.

**Synthesis of Co SAC/CNT-EM and FeOCl/CNT-M**

The Co SAC/CNT-EM was fabricated by coating a CNT-interwoven framework with the catalyst (Figure S37). Co/NOC-0.8 and carbon nanotubes (CNTs) were dispersed in a polyacrylonitrile (PAN) solution (0.1 wt% in 1-methyl-2-pyrrolidinone, NMP) at a 1:1 mass ratio to prepare a Co SAC-CNT suspension. CNTs were selected for their ability to form interwoven membrane structures that enhance permeability and electrical conductivity^[1]^. The mixture was ultrasonicated for 4 h in an ice bath, then vacuum-filtered through a commercial poly(tetrafluoroethylene) (PTFE) membrane (50 mm diameter, 0.45 μm pores) with a total loading of 2.4 mg cm^−2^. The resulting Co SAC/CNT electrocatalytic membrane (Co SAC/CNT-EM) was rinsed extensively with water and ethanol, followed by ambient drying. The resulting membranes are uniform and mechanically robust, with an effective area of 19.6 cm^2^.

FeOCl/CNT-M was fabricated via a two-step assembly: First, O-CNT Preparation: Oxidized CNTs (O-CNTs) were synthesized using a modified Hummers' method^[2]^. Second, FeOCl/CNT Formation: 100 mg O-CNTs were immersed in FeCl_3_ solution (prepared by dissolving 0.25 g FeCl_3_·6H_2_O in 20 mL ethanol), ultrasonicated, and stirred 1 h to adsorb iron ions. The mixture was dried and calcined (220 °C, 1 h, air), then washed with water/ethanol and dried at 60 °C.
FeOCl/CNT-M was subsequently prepared by substituting Co SAC and CNTs with pre-synthesized FeOCl/CNT in the aforementioned filtration procedure.

**Flow-through experiments**

As illustrated in Figure 5a, the electro-filtration module comprises a feed chamber equipped with a Ti mesh electrode as the anode (top), a permeate chamber containing an electrified membrane coated with Co SAC/CNT-EM serving as the cathode (middle), and a FeOCl/CNT membrane (FeOCl/CNT-M) acting as a downstream H_2_O_2_ activation layer (bottom). The FeOCl/CNT-M is tightly integrated with the Co SAC/CNT-EM to form a bilayer membrane, while a thin poly (tetrafluoroethylene) (PTFE) membrane inserted between the two layers as both a spacer and an insulating interface, thereby constructing the bilayer electrified membrane (Figures. S38–39).

Electrodes were positioned to maintain an electrolyte-filled gap between the electrodes, with flow-through mode preventing in-situ H_2_O_2_ oxidation at the upstream anode. The high cross-flow velocity (200 mL·min^−1^) maintained dissolved O_2_ supply for cathodic 2e^−^-ORR H_2_O_2_ generation. Experiments employed O_2_-saturated 0.1 M Na_2_SO_4_ recirculated via peristaltic pump (Masterflex, Cole-Parmer) at 25 °C. Permeate flux was regulated via needle valve (± 0.2 bar pressure gauge)^[1]^, with feed water traversing membrane pores under applied pressure to yield treated permeate. The system interfaced with a CHI 760E electrochemical workstation, applying –0.29 V vs. RHE to maximize H_2_O_2_ production based on catalyst optimization data.

**Energy consumption per order (EC_EO_)**

Energy consumption per order (EC_EO_) is the electrical energy required to reduce the concentration of pollutants by one order of magnitude per unit volume. EC_EO_ was calculated based on the following,

EC_EO_ (kWh·m^−3^·order^−1^) = (U_cell_ × I × t) / (V × log (C_0_/C_t_))

= (U_cell_ × I) / (1000 × Q× log (C_0_/C_t_))

where (U_cell_) is the average cell voltage across the reactor during operation. In this study, the flow-through reactor operated under a stable cell voltage of 2.0 V. (I) represents the experimentally recorded current throughout the long-term operation, and the average current was determined to be 1.65 × 10^−3^ A. (t) is the operation time (h), and (V) is the total treated solution volume (m^3^), and the flow rate (Q, m^3^·h^−1^) according to (Q = V / t). During the flow-through experiments, the average permeate flux remained stable at 1.01 mL·min^−1^, corresponding to Q = 6.078 × 10^−5^·m^3^·h^−1^. (C_0_) and (C_t_) correspond to the initial and final pollutant concentrations, respectively. Based on the long-term stability test, the average single-pass aniline removal efficiency reached 90%, corresponding to (log (C_0_/C_t_) = 1). Accordingly, the calculated energy consumption per order (EC_EO_) of the present system was determined to be 0.054 kWh·m^−3^·order^−1^.

**Computational methods**

We have employed the first-principles to perform all density functional theory (DFT) calculations within the generalized gradient approximation (GGA) using the Perdew-Burke-Ernzerhof (PBE) formulation^[3-5]^. We have chosen the projected augmented wave (PAW) potentials to describe the ionic cores and take valence electrons into account using a plane wave basis set with a kinetic energy cutoff of 450 eV^[6,7]^. Partial occupancies of the Kohn-Sham orbitals were allowed using the Gaussian smearing method with a width of 0.05 eV. The electronic energy was considered self-consistent when the energy change was smaller than 10^−5^ eV. A geometry optimization was considered convergent when the energy change was smaller than 0.01 eV·Å^−1^. The vacuum spacing in a direction perpendicular to the plane of the structure is 18 Å for the surfaces. The Brillouin zone integration is performed using 2×2×1 Monkhorst-Pack k-point sampling for a structure. Finally, the free energy was calculated using the equation:

G=E+ZPE-TS

where G, E, ZPE and TS are the free energy, total energy from DFT calculations, zero point energy and entropic contributions, respectively.

## Supplementary Note

**Note S1. H_2_O_2_-equivalent pathway deconvolution model analysis.**

**(1) Foundational methodological assumptions**

To semi-quantitatively elucidate the fate of electrogenerated H_2_O_2_ and distinguish the extent to which its apparent loss arises from secondary consumption or from an intrinsically suppressed 2e⁻-ORR, we established an H_2_O_2_-equivalent pathway deconvolution model. The mathematical validity and physical alignment of this semi-quantitative deconvolution framework are fundamentally sustained by the following two core physical-chemical postulates:

Assumption I: It is assumed that the global quantity of charge (*Q*) passed through the electrochemical system is entirely consumed by Faradaic charge-transfer reactions. Interfacial non-Faradaic processes, such as electric double-layer (EDL) charging or charging/discharging of background impurities, are considered negligible during the tracking period. Consequently, under a given total charge input *Q*, the maximum theoretical current efficiency toward the desired 2e⁻-ORR pathway is rigidly defined as 100%.

Assumption II: It is postulated that the secondary consumption of electrogenerated H_2_O_2_ is exclusively governed by two parallel pathways: H_2_O_2_ reduction reaction (HPRR) and chemical disproportionation reaction (HDR). Crucially, these secondary consumption channels are mechanistically independent of the intrinsic parasitic pathways occurring on the catalyst surface (e.g., 4e⁻-ORR). This kinetic independence permits the total intermediate molar flux and transferred electron flux to be mathematically partitioned via linear deconstruction and additivity.

**(2) Mathematical derivations and formulations**

Based on the postulate of exclusive Faradaic charge transfer (Assumption I), the total charge passed (*Q*) during the reaction is translated entirely into interfacial reaction flux. Accordingly, assuming the system undergoes the 2e⁻-ORR process with an absolute 100% selectivity to produce H_2_O_2_, the maximum theoretical molar yield of peroxide (*n*_H2O2, theoretical_) can be rigorously derived from Faraday’s law:

*n*_H2O2, theoretical_ = *Q* / 2F

where F is the Faraday constant (96,485 C·mol^−1^), and the factor of 2 accounts for the electron stoichiometry of peroxide formation from oxygen.

Furthermore, substantiated by the assumption of kinetic independence among the competing pathways (Assumption II), a closed-loop global molar balance equation can be established using *n*_H2O2, theoretical_ as a unified, normalized reference baseline. In a practical electrocatalytic system, the peroxide yield theoretically dictated by the total charge input is distributed among four distinct and mutually exclusive pathways:

*n*_H2O2, theoretical_ = *n*_H2O2, net_ + Δ*n*_HPRR_ + Δ*n*_HDR_ + *n*_4e⁻_

Where *n*_H2O2, net_ is the net molar accumulation of H_2_O_2_ successfully captured and quantified in the bulk electrolyte; Δ*n*_HPRR_ is the equivalent molar loss consumed via secondary HPRR within the active-site microenvironment; Δ*n*_HDR_ is the equivalent molar loss decomposed via secondary HDR on the catalyst surface; and *n*_4e⁻_ is the equivalent molar loss diverted to direct water formation via the parasitic 4e⁻-ORR channel due to incomplete intrinsic 2e⁻ selectivity.

To achieve standardized comparison across various catalyst configurations and operating conditions, both sides of the global molar balance equation are divided by *n*_H2O2, theoretical_, yielding the normalized percentage flux allocation equation:

100% = *P*_net_ + *P*_HPRR_ + *P*_HDR_ + *P*_4e⁻_

**Note S2. Experimental execution and stepwise kinetic deconvolution calculations.**

**(1) Experimental assumptions and control conditions**

The methodological validity of this semi-quantitative analysis strictly relies on the following four foundational experimental assumptions and control conditions:

i). Under N_2_ atmosphere with externally added H_2_O_2_ and an applied operational potential, no appreciable new H_2_O_2_ generation occurs. Therefore, the measured peroxide loss under these biased conditions can be reasonably and exclusively attributed to the coupled consumption pathways of HPRR and HDR.

ii) Under N_2_ atmosphere with externally added H_2_O_2_ and in the absolute absence of an applied potential, interfacial electrochemical reduction is assumed to be completely blocked. Consequently, the measured peroxide loss under these open-circuit conditions is assumed to originate primarily from the HDR pathway.

iii) The subsequent kinetic fate of in-situ electrogenerated peroxide trapped within the catalyst layer can be simulated, to a first-order approximation, by the exogenous introduction of H_2_O_2_.

iv) All control experiments should be conducted under identical catalyst structures and mass-transfer conditions.

**(2) Standardized experimental configurations and procedures**

Guided by the control conditions established above, the experimental operations were systematically executed according to the following protocols:

All electrocatalytic and heterogeneous catalytic evaluations were performed in a customized H-type glass cell (50 mL per compartment) partitioned by a proton exchange membrane. A 0.1 M Na_2_SO_4_ aqueous solution (pH = 5.73) served as the standard electrolyte. All catalysts across the loading spectrum were uniformly deposited onto identical hydrophilic carbon paper substrates with a fixed loading of 1 mg·cm^−2^ and a geometric area of 2 × 2 cm^2^ (total catalyst mass: 4 mg).

The H_2_O_2_ output test was conducted in O_2_-saturated Na_2_SO_4_ electrolyte, whereas the HPRR and HDR consumption tests were performed in N_2_-saturated Na_2_SO_4_ electrolyte. The added H_2_O_2_ was introduced into the cathodic compartment of a typical three-electrode H-cell. The initial H_2_O_2_ concentration was set at 500 μmol·L^−1^ for Co-free NOC, 600 μmol·L^−1^ for Co/NOC-0.3, Co/NOC-0.5 and 800 μmol·L^−1^ for Co/NOC-0.8, Co/NOC-2.0, Co/NOC-3.8, based on the following calculations.

Throughout the reaction, the applied operational potential was constantly maintained at −0.29 V (or kept at open-circuit potential for the independent HDR tracking). After a continuous reaction interval of exactly 1 h, the remaining H_2_O_2_ in the system was sampled and quantitatively determined by the TPO-UV-vis method.

**(3) Stepwise matrix deconvolution calculations**

Based on the experimental assumptions and configurations established above, a three-step matrix extraction was applied to mathematically isolate the overlapping kinetic pathways:

**i). Step I: Calculation of HDR**

Based on experimental condition under N_2_ atmosphere with externally added H_2_O_2_, the peroxide loss measured under open-circuit conditions (OCP) yields the independent rate of chemical disproportionation:

Δ*n*_HDR_ = *n*_0_ − *n*_HDR_

Where *n*_0_ is the initial amount of added H_2_O_2_; *n*_HDR_ represents the residual moles of H_2_O_2_ after 1 h.

**ii). Step II: Deconvolution of HPRR**

Based on experimental condition under N_2_ atmosphere with externally added H_2_O_2_ and an applied operational potential, the total H_2_O_2_ loss (Δ*n*_potential-nitrogen_) captures the simultaneous execution of both parasitic pathways:

Δ*n*_potential-nitrogen_ = *n*_0_ − *n*_potential-nitrogen_ = Δ*n*_HPRR_ + Δ*n*_HDR_

Where *n*_potential-nitrogen_ is the remaining moles of H_2_O_2_ under potential condition;

By subtracting the independently isolated chemical decomposition baseline (Δ*n*_HDR_) from Step I, the net molar loss allocated to the electrochemical reduction (HPRR) channel is precisely decoupled:

Δ*n*_HPRR_ =Δ*n*_potential-nitrogen_ − Δ*n*_HDR_

**iii). Step III: Normalization**

Finally, utilizing the common equivalent reference baseline derived from the total charge input (Q) passed during the real O_2_-ORR test (as established in **Note S1**), the distinct molar loss values were converted into normalized percentage contributions relative to the baseline maximum theoretical H_2_O_2_ yield (*n*_H2O2, theoretical_):

*P*_net_ = 100× *n*_H2O2, net_ / *n*_H2O2, theoretical_

*P*_HPRR_ = 100× Δ*n*_HPRR_ / *n*_H2O2, theoretical_

*P*_HDR_ = 100× Δ*n*_HDR_ / *n*_H2O2, theoretical_

The percentage allocation of the direct 4e⁻-ORR pathway (*P*_4e⁻_) is subsequently extracted as a mathematical residual fraction that cannot be attributed to net H_2_O_2_ accumulation or secondary consumption pathways:

*P*_4e⁻_ = 100% − *P*_net_ − *P*_HPRR_ − *P*_HDR_

The detailed stepwise calculations for each catalyst configuration are tabulated in **Table S4** and **Table S5**.

**Note S3. Methodological boundaries and limitations**

Although the H_2_O_2_-equivalent pathway deconvolution model provides a practical semi-quantitative framework for evaluating peroxide generation and consumption pathways, several limitations should be acknowledged.

(1) The decoupling of HPRR and HDR is based on independently measured control experiments under applied-potential and open-circuit conditions. Therefore, potential dynamic coupling between electrochemical reduction and catalytic disproportionation under real ORR operation is approximated as additive contributions.
(2) The model treats externally introduced H_2_O_2_ as a first-order approximation of electrogenerated peroxide. Possible differences in local concentration distribution, transient surface accumulation, and interfacial mass-transfer behaviour are not explicitly incorporated.
(3) This methodology is primarily intended for comparative evaluation among catalyst systems measured under identical experimental configurations and should not be interpreted as an absolute quantification of all reaction pathways across different reaction environments.

## 2. Supplementary Figures

**Figure S1.** SEM images of the polymerized two-dimensional nanosheet precursor of the Co SACs.

**Figure S2.** SEM images of Co SAC (Co/NOC-0.8).

**Figure S3.** (a) SEM images of Co SAC (Co/NOC-0.8). (b-d) TEM images of Co SACs (Co/NOC-0.8, Co/NOC-2.0, Co/NOC-3.8).

**Figure S4.** STEM-EDS elemental mapping of Co/NOC-0.8.

**Figure S5.** XRD patterns of series Co SACs.

**Figure S6.** N 1s XPS spectra of (a) Co/NOC-0.8, (b) Co/NOC-2.0 and (c) Co/NOC-3.8 electrocatalysts.

**Figure S7.** Co 2p XPS spectra of (a) Co/NOC-0.8, (b) Co/NOC-2.0 and (c) Co/NOC-3.8 electrocatalysts.

**Figure S8.** X-ray near edge structure (XANES) spectra.

**Figure S9.** (a) FT-EXAFS R-space fitting curves of Co/NOC-0.8. (b) The corresponding FT-EXAFS k space fitting curves of Co in Co/NOC-0.8.

**Figure S10.** (a) FT-EXAFS R-space fitting curves of Co/NOC-2.0. (b) The corresponding FT-EXAFS k space fitting curves of Co in Co/NOC-2.0.

**Figure S11.** (a) FT-EXAFS R-space fitting curves of Co/NOC-3.8. (b) The corresponding FT-EXAFS k space fitting curves of Co in Co/NOC-3.8.

**Figure S12.** (a) FT-EXAFS R-space fitting curves of Co/NOC-0.8-unoxidised. (b) The corresponding FT-EXAFS k space fitting curves of Co in Co/NOC-0.8-unoxidised.

**Figure S13.** (a) FT-EXAFS R-space fitting curves of Co/NOC-3.8-unoxidised. (b) The corresponding FT-EXAFS k space fitting curves of Co in Co/NOC-3.8-unoxidised.

**Figure S14.** (a)-(c) X-ray near edge structure (XANES) spectra of series Co SACs. (d) The corresponding k^3^ -weighted Fourier transform (FT) EXAFS spectra.

**Figure S15.** Raman spectra of series Co SACs.

**Figure S16.** EPR spectra of Co SACs.

**Figure S17.** (a) C k-edge XANES spectra of Co/NOC-0.8-unoxidised and Co/NOC-0.8. (b) Expanded region 1 and 2 in (a).

The spectacle exhibits three characteristic peaks corresponding to π* (C=C), π* (C=O), and σ* (C=C) transitions^[8,9]^. The characteristic peak at 286.2 eV (π* (C=C), attributed to the electron transition of C 1s to unoccupied π* state) could reflect the integrity level of the *sp^2^*-hybridized carbon configuration^[10]^. Compared with unoxidised counterpart, Co/NOC-0.8 displays a decreased π* (C=C) peak intensity, suggesting a lower degree of graphitization likely caused by carbon atom rearrangement and the introduction of vacancy defects^[11,12]^. In addition, a negative shift of approximately 0.2 eV in the σ* (C=C) peak (assigned to the electron transition of C 1s to the unoccupied σ* state), indicates the generation of intrinsic sp^3^-hybridized carbon defects after oxidation^[13,14]^.

**Figure S18.** C 1s XPS spectra of (a) Co/NOC-0.8-unoxidised, (b) Co/NOC-0.8, (c) Co/NOC-2.0-unoxidised, (d) Co/NOC-2.0, (e) Co/NOC-3.8-unoxidised and (f) Co/NOC-3.8 electrocatalysts.

The C 1s X-ray photoelectron spectroscopy (XPS) spectra can be deconvoluted into three peaks at 284.8, 286.6, and 288.4 eV, corresponding to *sp^2^* C, *sp^3^* C, and C-N/O species, respectively^[8]^. Notably, the relative sp^3^-hybridized carbon content in Co/NOC-0.8 increases from 17.5% to 19.5% compared with that of unoxidised counterpart, and a similar trend is observed for Co/NOC-2.0 and Co/NOC-3.8, indicating the generation of additional intrinsic sp^3^-type defects after HNO_3_ oxidation^[9]^.

**Figure S19.** Structural model and formation energy (E_f_) of Co-N_2_O_2_.

**Figure S20.** Structural models and formation energies (E_f_) of Co-N_2_O_2_ with different second-shell vacancy defects.

**Figure S21.** Corresponding H_2_O_2_ selectivity.

**Figure S22.** Representative TEM and HAADF-STEM images together with corresponding EDS elemental mapping (C, N, O, and Co) of the Co/NOC-0.3 sample. Isolated bright spots observed in the HAADF-STEM image indicate the presence of atomically dispersed Co species.

**Figure S23.** Representative TEM and HAADF-STEM images together with corresponding EDS elemental mapping (C, N, O, and Co) of the Co/NOC-0.5 sample. Isolated bright spots observed in the HAADF-STEM image indicate the presence of atomically dispersed Co species.

**Figure S24.** (a) LSV curves and corresponding H_2_O_2_ detection currents recorded at the ring electrode for Co/NOC-3.8 at different rotation rates in O_2_-saturated 0.1 M Na_2_SO_4_. (b) Corresponding H_2_O_2_ selectivity.

**Figure S25.** Evaluation of electrocatalytic H_2_O_2_ production performance and experimental setup. (a) Current response. (b) H_2_O_2_ yield at different applied potentials; −0.29 V was selected as the applied potential for evaluating different catalysts. (c) Photograph of the typical H-type electrochemical cell setup.

**Figure S26.** ORR polarization curves in 0.1 M Na_2_SO_4_ before and after the addition of 10 mM KSCN.

**Figure S27.** (a) LSV curves and corresponding H_2_O_2_ detection currents recorded at the ring electrode in O_2_-saturated 0.1 M Na_2_SO_4_. (b) Corresponding H_2_O_2_ selectivity.

**Figure S28.** Photograph of the in-situ ATR-FTIR instrument.

**Figure S29.** In situ ATR-FTIR spectra of (a) Co-free NOC catalyst and (b) Co-NOC-3.8 in O_2_-saturated 0.1 M Na_2_SO_4_ over a potential range from 0.1 to –0.7 V.

**Figure S30.** In situ ATR-FTIR spectra of (a) Co-free NOC and (b) Co/NOC-0.8 in N_2_-saturated 0.1 M Na_2_SO_4_ containing 10 mM H_2_O_2_ over a potential range from 0.1 to –0.7 V.

**Figure S31.** In situ ATR-FTIR spectra of Co-free NOC in 0.1 M Na_2_SO_4_ containing 10 mM H_2_O_2_.

**Figure S32.** The structure models of 1-Co sample and the corresponding theoretical Co loadings.

**Figure S33.** The structure models of 2-Co sample and the corresponding theoretical Co loadings.

**Figure S34.** The structure models of 4-Co sample and the corresponding theoretical Co loadings.

**Figure S35.** Correlation between Co single-site density and d-band center and Bader charge in 1-Co, 2-Co and 4-Co models.

**Figure S36.** Adsorption energy of H_2_O_2_ on the Co sites in 1-Co, 2-Co, 4-Co.

**Figure S37.** Schematic illustration of the fabrication procedure of the Co SAC/CNT-EM.

**Figure S38.** Photograph of the self-designed flow-through electrified membrane filtration system.

**Figure S39.** Photograph of the as-fabricated bilayer membrane.

**Figure S****40.** Calculation of the collection efficiency. (a) LSV curves in 1 M KCl with 10 mM K_3_[Fe(CN)_6_] at different rotation speeds. (b) Calculation of the collection efficiency (N).

## 3. Supplementary Tables

**Table S1.** Co loading for different samples determined by ICP-MS.

| **Sample** | **Co loading (wt.%)** |
| --- | --- |
| Co/NOC-0.3 | 0.27 |
| Co/NOC-0.5 | 0.46 |
| Co/NOC-0.8 | 0.84 |
| Co/NOC-2.0 | 1.96 |
| Co/NOC-3.8 | 3.83 |
| Co/NOC-0.8-unoxidised | 0.94 |
| Co/NOC-2.0-unoxidised | 2.34 |
| Co/NOC-3.8-unoxidised | 5.10 |

**Table S2.** EXAFS fitting parameters at the Co-K-edge for the various samples. (S_0_^2^=0.95 for Co)

| Sample | Shell | *CN^a^* | R (Å)^b^ | σ^2^(Å^2^)^c^ | ΔE_0_(eV)^d^ | *R* factor |
| --- | --- | --- | --- | --- | --- | --- |
| Co foil | Co-Co | 12.0 | 2.49 | 0.0060 | 7.3 | 0.002 |
| Co Pc | Co-N | 4.0 | 1.91 | 0.0010 | 6.3 | 0.016 |
| Co/NOC-0.8-unoxidised | Co-N | 2.2 | 1.93 | 0.0086 | -5.5 | 0.007 |
|  | Co-O | 2.0 | 2.08 | 0.0038 |  |  |
| Co/NOC-3.8-unoxidised | Co-N | 2.0 | 1.90 | 0.0016 | -1.2 | 0.009 |
|  | Co-O | 2.0 | 2.05 | 0.0018 |  |  |
| Co/NOC-0.8 | Co-N | 1.92 | 1.92 | 0.0091 | -5.3 | 0.007 |
|  | Co-O | 2.07 | 2.07 | 0.0091 |  |  |
| Co/NOC-2.0 | Co-N | 2.0 | 1.94 | 0.0090 | 7.8 | 0.010 |
|  | Co-O | 1.8 | 2.10 | 0.0034 |  |  |
| Co/NOC-3.8 | Co-N | 2.3 | 1.92 | 0.0080 | -4.2 | 0.010 |
|  | Co-O | 2.0 | 2.09 | 0.0053 |  |  |

^a^CN, coordination number; ^b^R, the distance to the neighboring atom; ^c^σ^2^, the Mean Square Relative Displacement (MSRD); ^d^ΔE_0_, inner potential correction; R factor indicates the goodness of the fit. S_0_^2^ was fixed to 0.95, according to the experimental EXAFS fit of Co foil by fixing CN as the known crystallographic value. A reasonable range of EXAFS fitting parameters: 0.700 < Ѕ_0_^2^ < 1.000; CN > 0; σ^2^(Å^2^) > 0; |ΔE^0^| < 10 eV; R factor < 0.02.

**Table S3.** EXAFS fitting parameters at the Co K-edge for different structural models. (S_0_^2^=0.95 for Co)

| Sample | Shell | *CN^a^* | R (Å)^b^ | σ^2^(Å^2^)^c^ | ΔE_0_(eV)^d^ | *R* factor |
| --- | --- | --- | --- | --- | --- | --- |
| A | Co-N | 2.2 | 1.93 | 0.0086 | -5.5 | 0.007 |
|  | Co-O | 2.0 | 2.08 | 0.0038 |  |  |
| B | Co-N | 2.1 | 1.89 | 0.0076 | -4.7 | 0.008 |
|  | Co-O | 2.1 | 1.98 | 0.0045 |  |  |
| C | Co-N | 2.0 | 1.93 | 0.0069 | -5.7 | 0.009 |
|  | Co-O | 2.1 | 1.96 | 0.0037 |  |  |
| A-1 | Co-N | 1.92 | 1.92 | 0.0091 | -5.3 | 0.007 |
|  | Co-O | 2.07 | 2.07 | 0.0091 |  |  |
| A-2 | Co-N | 2.2 | 1.93 | 0.0085 | -6.1 | 0.010 |
|  | Co-O | 1.9 | 2.11 | 0.0045 |  |  |
| A-3 | Co-N | 1.9 | 1.96 | 0.0093 | -5.9 | 0.011 |
|  | Co-O | 2.2 | 2.14 | 0.0085 |  |  |
| A-4 | Co-N | 1.9 | 1.91 | 0.0093 | -5.4 | 0.010 |
|  | Co-O | 2.2 | 2.05 | 0.0097 |  |  |
| A-5 | Co-N | 2.0 | 1.88 | 0.0094 | -5.5 | 0.009 |
|  | Co-O | 2.1 | 2.03 | 0.0085 |  |  |

The proposed structural models are shown in Figures S19 and S20. ^a^CN, coordination number; ^b^R, the distance to the neighboring atom; ^c^σ^2^, the Mean Square Relative Displacement (MSRD); ^d^ΔE_0_, inner potential correction; R factor indicates the goodness of the fit. S_0_^2^ was fixed to 0.95, according to the experimental EXAFS fit of Co foil by fixing CN as the known crystallographic value. A reasonable range of EXAFS fitting parameters: 0.700 < Ѕ_0_^2^ < 1.000; CN > 0; σ^2^(Å^2^) > 0; |ΔE^0^| < 10 eV; R factor < 0.02.

**Table S4.** Semi-quantitative deconvolution of ideal H_2_O_2_-equivalent output into net H_2_O_2_ accumulation, HPRR consumption, HDR consumption, and 4e⁻-ORR pathway contribution for the various Co SAC samples.

| Sample | *n*_H2O2, net_ (μmol·L^−1^·h^−1^) | *n*_H2O2, theoretical_ (μmol·L^−1^·h^−1^) | Δ*n*_potential-nitrogen_ (μmol·L^−1^·h^−1^) | Δ*n*_HPRR_ (μmol·L^−1^·h^−1^) | Δ*n*_HDR_ (μmol·L^−1^·h^−1^) | *n*_4e⁻_ (μmol·L^−1^·h^−1^) |
| --- | --- | --- | --- | --- | --- | --- |
| Co-free NOC | 54.30 | 497.22 | 65.01 | 44.24 | 20.77 | 377.91 |
| Co/NOC-0.3 | 286.67 | 570.17 | 69.34 | 43.58 | 25.76 | 214.16 |
| Co/NOC-0.5 | 305.17 | 576.69 | 67.75 | 40.25 | 27.50 | 203.77 |
| Co/NOC-0.8 | 640.30 | 813.11 | 42.63 | 30.79 | 11.84 | 130.17 |
| Co/NOC-2.0 | 424.10 | 780.18 | 81.60 | 62.67 | 18.93 | 274.48 |
| Co/NOC-3.8 | 254.52 | 814.30 | 139.38 | 83.88 | 55.49 | 420.39 |

**Table S5.** Contribution to ideal H_2_O_2_-equivalent output (%).

| Sample | *P*_net_ (%) | *P*_theoretical_ (%) | *P*_HPRR_ (%) | *P*_HDR_ (%) | *P*_4e⁻_ (%) |
| --- | --- | --- | --- | --- | --- |
| Co-free NOC | 10.92 | 100 | 8.90 | 4.18 | 76.00 |
| Co/NOC-0.3 | 50.27 | 100 | 7.64 | 4.52 | 37.57 |
| Co/NOC-0.5 | 52.92 | 100 | 6.98 | 4.77 | 35.33 |
| Co/NOC-0.8 | 78.75 | 100 | 3.79 | 1.46 | 16.01 |
| Co/NOC-2.0 | 54.36 | 100 | 8.03 | 2.43 | 35.18 |
| Co/NOC-3.8 | 31.26 | 100 | 10.30 | 6.81 | 51.63 |

**Table S6.** ICP-MS analysis of metal leaching after long-term operation of the electrified membrane system.

| **Elements** | **Concentration** (μg·L^−1^) |
| --- | --- |
| Co | 16.02 |
| Fe | 15.19 |

**Table S7.** Comparison of pollutant removal performance and energy consumption with representative advanced electro-Fenton systems reported in recent years.

| Catalysts/Materials | pH | Reactor Type | Pollutants | Stability | Energy consumption (kWh·m^−3^·order^−1^) | Reference |
| --- | --- | --- | --- | --- | --- | --- |
| **Co/NOC-0.8, FeOCl** | **5.7** | **Flow-through membrane reactor** | **Aniline** | **72h continuous** | **0.054** | **This work** |
| Cu_SAs/ACs_-NC/CMM | 6.5 | Flow-through membrane reactor | Tetracycline | 4 cycles, 2h each | 0.102 | [15] |
| AuNCs | 6.7 | Flow-through membrane reactor | Tetracycline | 4 cycles, 2h each | 0.25 | [16] |
| FeOCl-CNT | 6.5 | Flow-through membrane reactor | Tetracycline | / | 0.336 | [17] |
| Stainless-steel | 7 | Flow-through  plate electrode | Carbamazepine | 30h continuous | 4.20 | [18] |
| CoCu-DACs-CN | 3 | Flow cell | BPA | 20h continuous | 0.33 | [19] |
| Ce-doped Ti/PbO_2_ | 7 | Single-cell | Dibutyl phthalate | / | 1.20 | [20] |
| P-doped Cu_2_PO_4_/EGF | 6-9 | Single-cell | Sulfamethoxazole | 5 cycles, 2h each | 0.269 | [21] |
| Ce/Fe@NPC-GF | 3 | Single-cell | Sulfamethoxazole | 8 cycles, 2h each | 0.636 | [22] |

## 4. References

[1]	Y. Fan, Y. Yan, O. Nwokonkwo, D. J. Rivera, W. Pan, E. Chen, J.-Y. Kim, J. Simon, M. Saffer-Meng, X. Wang, C. Muhich, L. R. Winter, *Nat. Chem. Eng.* **2025**, *2*, 379–390.

[2]	S. Zhong, Z.-S. Zhu, P. Zhou, L. Shi, X. Duan, B. Lai, S. Wang, *Appl. Nano Mater.* **2022**, *5*, 12095–12106.

[3]	G. Kresse, J. Furthmüller, *Comput. Mater. Sci*. **1996**, *6*, 15–50.

[4]	G. Kresse, J. Furthmüller, *Phys. Rev. B* **1996**, *54*, 11169–11186.

[5]	J. P. Perdew, K. Burke, M. Ernzerhof, *Phys. Rev. Lett*. **1996**, *77*, 3865–3868.

[6]	G. Kresse, D. Joubert, *Phys. Rev. B* **1999**, *59*, 1758–1775.

[7]	P. E. Blöchl, *Phys. Rev. B* **1994**, *50*, 17953–17979.

[8]	K. Yu, S. Guan, W. Zhang, W. Zhang, Y. Meng, H. Lin, Q. Gao, *Angew. Chem. Int. Ed.* **2025**, *64*, e202502383.

[9]	Z. Liu, B. Li, Y. Feng, D. Jia, C. Li, Y. Zhou, *Small Methods* **2022**, *6*, 2270059.

[10]	S. Liu, Y. Zhang, B. Ge, F. Zheng, N. Zhang, M. Zuo, Y. Yang, Q. Chen, *Adv. Mater.* **2021**, *33*, 2103133.

[11]	Y. Dong, Q. Zhang, Z. Tian, B. Li, W. Yan, S. Wang, K. Jiang, J. Su, C. W. Oloman, E. L. Gyenge, R. Ge, Z. Lu, X. Ji, L. Chen, *Adv. Mater.* **2020**, *32*, 2001300.

[12]	X. Wang, Y. Jia, X. Mao, L. Zhang, D. Liu, L. Song, X. Yan, J. Chen, D. Yang, J. Zhou, K. Wang, A. Du, X. Yao, *Chem* **2020**, *6*, 2009–2023.

[13]	W. Wang, L. Shang, G. Chang, C. Yan, R. Shi, Y. Zhao, G. I. N. Waterhouse, D. Yang, T. Zhang, *Adv. Mater.* **2019**, *31*, 1808276.

[14]	Y. Li, J. Chen, Y. Ji, Z. Zhao, W. Cui, X. Sang, Y. Cheng, B. Yang, Z. Li, Q. Zhang, L. Lei, Z. Wen, L. Dai, Y. Hou, *Angew. Chem. Int. Ed.* **2023**, *62*, e202306491.

[15]	Z. Zhang, G. Huang, Y. Li, Y. Yao, X. Chen, Y. Zhang, Y. Luo, K. Zhao, *Chem. Eng. J.* **2025**, *515*, 163677.

[16]	F. Liu, Y. Liu, Q. Yao, Y. Wang, X. Fang, C. Shen, F. Li, M. Huang, Z. Wang, W. Sand, J. Xie, *Environ. Sci. Technol.* **2020**, *54*, 5913–5921.

[17]	Z. Li, C. Shen, Y. Liu, C. Ma, F. Li, B. Yang, M. Huang, Z. Wang, L. Dong, S. Wolfgang, *Appl. Catal. B: Environ.* **2020**, *260*, 118204.

[18]	Y. Duan, D. L. Sedlak, *Environ. Sci. Technol.* 2024, *58*, 14042–14051.

[19]	W. Sun, G. Liu, H. Zou, S. Wang, X. Duan, S. You, *Angew. Chem. Int. Ed*. **2025**, *65*, e19275.

[20]	D. Deng, X. Wu, M. Li, S. Qian, B. Tang, S. Wei, J. Zhang, *Chemosphere* **2020**, *259*, 127488.

[21]	Z. Yan, H. Qi, X. Shi, Z. Liu, Z. Sun, *Sep. Purif. Technol.* **2024**, *339*, 126716.

[22]	S. Qiu, Y. Wang, J. Wan, Y. Ma, Z. Yan, S. Yang, *Chemosphere* **2021**, *273*, 130269.

## 5. Author contributions

J.C., W.R. and X.D. designed the research. J.C. carried out the experiments and wrote the manuscript. W.R. and X.D. supervised the research and further revised the paper. H.Z., Q.Z. and S.Z. performed data analysis and some characterizations. L.S., L.G. and S.W. modified the manuscript. All authors contributed to the discussion of the manuscript.
